# The proteomic landscape of trophoblasts unravels calcium-dependent syncytialization processes and beta-chorionic gonadotropin (ß-hCG) production

**DOI:** 10.1186/s12958-025-01362-7

**Published:** 2025-03-04

**Authors:** Anna-Lena Gehl, Daniel Klawitter, Ulrich Wissenbach, Marnie Cole, Christine Wesely, Heidi Löhr, Petra Weissgerber, Adela Sota, Markus R. Meyer, Claudia Fecher-Trost

**Affiliations:** Experimental and Clinical Pharmacology and Toxicology, Center for Molecular Signaling (PZMS), Saarland University, Buildings 61.4 and 46, 66421 Homburg, Germany

**Keywords:** Placenta, Trophoblast, BeWo cells, Syncytialization, Hormone synthesis, Calcium, Proteome

## Abstract

**Background:**

The syncytiotrophoblast (STB) layer of the placenta is formed by cell fusion of cytotrophoblasts, acts as a feto-maternal barrier, is required for the production of pregnancy hormones such as chorionic gonadotropin, estradiol and progesterone and is also responsible for feto-maternal mineral exchange such as calcium. Adequate mineral supply and placental hormone production are essential for the maintenance of pregnancy, and disturbances in trophoblast integrity are associated with pregnancy complications. Since knowledge about the identity and expression levels of proteins in trophoblast and syncytiotrophoblast cells is limited so far, we analyzed the proteomes of trophoblast-like and syncytiotrophoblast-like BeWo cells under different calcium conditions. The investigation of protein expression profiles in combination with hormone assays can provide a better understanding of calcium-dependent cellular processes in trophoblasts and syncytiotrophoblasts.

**Methods:**

Here, we combine human trophoblast model cell cultures, hormone assays, antibody-based detection methods and high-resolution mass spectrometry analyzes to assess changes in cellular processes during syncytialization.

**Results:**

We monitored the changes in protein expression profiles during forskolin induced syncytialization of trophoblast-like cells in an unbiased manner and show that the expression of numerous proteins is strongly altered. Among them are enzymes of the glucocorticoid and sex hormones synthesis pathways such as cytochrome P450 (CYP) 19A1, CYP11A1, adrenodoxin (FDX1), hydroxysteroid dehydrogenase (HSD) 11β2 and HSD17β1, whose expression is strongly induced by syncytialization. The production of beta human chorionic gonadotropin (ß-hCG), progesterone and estradiol increase during syncytialization, while the secretion and synthesis of ß-hCG and the expression of several protein syncytiotrophoblast markers show a clear calcium dependence.

**Conclusion:**

The broad applicability of semi-quantitative proteome profiling of cytotrophoblast- and syncytiotrophoblast-like cells provides new insights into signaling processes that occur in cytotrophoblasts /syncytiotrophoblasts during pregnancy.

**Supplementary Information:**

The online version contains supplementary material available at 10.1186/s12958-025-01362-7.

## Background

The human placenta is a highly specialized organ necessary for the normal growth and development of the fetus. Therefore, the proper differentiation and fusion of villous cytotrophoblasts into multinucleated syncytiotrophoblasts (STB) plays a crucial role in implantation, feto-maternal nutrient exchange, and the production of pregnancy hormones as human chorionic gonadotropin (hCG), progesterone and estrogen [1–5]. However, an insufficient syncytialization process is associated with various pregnancy disorders as pre-eclampsia (PE), intrauterine growth restriction (IUGR) or spontaneous abortion [1, 6, 7]. The involvement of several proteins in syncytialization has been investigated in various studies. For example, syncytin-1 [1–4, 7], syncytin-2 [2, 4, 7], E-cadherin [6, 7] or the formation of hCG [3, 6, 8, 9] are common syncytialization markers of trophoblasts.

The syncytialization process is mediated by several signaling pathways including cAMP-PKA, MAPK11/14, ERK /2, Wnt/beta-catenin and p38MAPK [1, 2, 5–7, 10]. Activation of the cAMP-PKA pathway leads to the formation of the transcription factor Glial Cell Missing 1 (GCM1), which initiates the expression of Syncytin-1 (ERVW-1), which is essential for cell fusion [1–5, 7]. In addition, an increase in cyclic adenosine monophosphate (cAMP) promotes the expression of marker proteins such as hCG β-subunit, placental protein 13, human placental lactogen and placental alkaline phosphatase [5].

Primary trophoblasts survive for a few days in culture, therefore in vitro cell culture models for human trophoblasts, such as BeWo or JEG-3 cells, have been used for many years [2–4, 6, 8, 11, 12]. BeWo cells originate from a chorionic carcinoma of the fetal placenta derived from a male fetus and have similar properties to primary trophoblasts. BeWo cells grow adherently, are epithelial-like and syncytialization is inducible by forskolin (FSK). Like the primary trophoblasts of the human placenta, BeWo cells also produce and secrete hormones, including the peptide hormones human chorionic gonadotropin (hCG = pregnancy hormone) and chorionic somatomammotropin (placental lactogen) as well as various steroid hormones such as progesterone and estradiol. In contrast, JEG-3 cells do not syncytialize after FSK stimulation [13]. BeWo cells are therefore a suitable model system to investigate trophoblast syncytialization and hormone production and secretion.

The active transport of minerals from the maternal to the fetal side of the placenta is achieved by the syncytiotrophoblast layer [9, 14–17]. Some ion channels of the transient receptor potential (TRP) family appear to play an essential role in this process. Among others TRPV6, TRPV2 and TRPM7 transcripts are detectable in human syncytiotrophoblasts and mouse placenta [15, 17–20]. An insufficient supply of calcium to the fetus during pregnancy manifests itself as skeletal dysplasia accompanied with reduced growth of the embryo. This phenotype was observed in TRPV6 knockout (*Trpv6*^*−/−*^) mice as well as in *Trpv6*^*mt/mt*^ mice which have a single point mutation within the pore of the channel. Compared to wild-type trophoblasts, *Trpv6*^*mt/mt*^ trophoblasts have a lower calcium content, different morphology and altered protein profiles, e.g. proteins that are necessary for the formation of the extracellular matrix are deregulated [19, 21]. Additionally primary trophoblasts from wildtype mice cultured in the presence of low calcium show a phenotype similar to *Trpv6*^*−/−*^ mice [19]. This suggests that the role of calcium should also be further investigated in human trophoblasts and syncytiotrophoblasts. Human BeWo cells also express TRPV2 and TRPV6 [22, 23] and their morphology also depend on the extracellular calcium concentration [22]. The morphology and physiology of mouse and human placenta differ in many aspects, e.g. there appears to be no orthologue of the human pregnancy hormone β-hCG in mice. Therefore, we decided to analyze the calcium dependence of protein profiles in BeWo cells, which have many of the characteristics of human placental trophoblasts and therefore represent cytotrophoblast- and, after FSK induced cell fusion, syncytiotrophoblast-like phenotypes.

## Materials and methods

### BeWo cell culture

Human BeWo cells (Ubigene, Montrouge, France) were cultured in F-12 Nut Mix medium (ThermoFisher, Karlsruhe, Germany) containing 2mM Glutamax, 20% fetal bovine serum (Corning, Tewksbury, MA, USA) and 1% penicillin/streptomycin solution (Sigma-Aldrich-Merck, Darmstadt, Germany) in 75cm^2^ culture flasks in presence of 5% CO_2_ at 37 °C. The medium was changed every two days and at 90% confluence the cells were washed with PBS buffer (136mM NaCl, 2.7mM KCl, 1.47mM KH_2_PO, 8mM Na_2_HPO_4_ × 2H_2_O) before trypsin incubation (21.5µM Trypsin, 684µM EDTA in PBS).

### Immunostaining of BeWo cells

The BeWo cells were grown in a 24 well plate with poly-L-lysine coated glass coverslips (diameter 12 mm) until 70% confluence. To induce syncytialization, 2mL cell culture medium was supplemented with 0.45% DMSO (Sigma-Aldrich-Merck, Darmstadt, Germany) or 30µM FSK/0.45% DMSO (Sigma-Aldrich, Darmstadt, Germany) and incubated for 48 h. The medium was removed, and the cells were washed with 300 µl PBS and fixed with methanol (-20 °C) for 20 min. Cells were washed with PBS, 300 µl of freshly prepared blocking buffer [0.1% Triton X (Carl Roth, Karlsruhe, Germany), 1% normal donkey serum (Sigma-Aldrich-Merck, Darmstadt, Germany) and 3% BSA (Carl Roth, Karlsruhe, Germany) in PBS was applied and the cells were incubated for 1 h at room temperature on the shaking plate. After removing the blocking buffer, the cells were washed 3 times with PBS for three minutes and were incubated over night at 4 °C with 250 µl of the zona occludens protein (ZO-1) specific primary antibody from rabbit (1:1000 in blocking buffer, Invitrogen, Schwerte, Germany). The next day, the primary antibody was removed, the cells were washed 3 times with PBS and incubated for 1 h at RT, protected from light, with 300 µl of the secondary antibody Alexa Fluor 488 anti-rabbit antibody (Invitrogen, Schwerte, Germany, 1:1000 in blocking buffer). After removal of the antibody and a washing step, the cell nuclei were stained using DAPI (2 µg/ml in PBS, Sigma-Aldrich-Merck, Darmstadt, Germany), the cells were washed, and the coverslips were attached to slides using mounting agent (Immu-Mount^™^, Thermo Scientific). Images of the stained BeWo cells were taken with an Imager 2 microscope (Zeiss) obtaining a Axiocam MRm (Zeiss). The fusion index was analyzed (Axio Vision 4.8) by calculating the cell fusion normalized to the cell number using the formula ((NNS-S)/T) x 100% (NNS = number of nuclei in syncytia, S = number of syncytia, T = total number of nuclei; 0.94mM Ca^2+^ DMSO *N* = 6, *n* = 508; 0.94mM Ca^2+^ FSK *N* = 6, *n* = 348; 0.35mM Ca^2+^ DMSO *N* = 6, *n* = 367; 0.35mM Ca^2+^ FSK *N* = 6, *n* = 256).

### Determination of β-hCG, estrogen and progesterone in BeWo cell culture supernatant

To determine hormone production before/after FSK incubation, small cell culture flasks (Falkon, 25cm^2^) were cultured until cells reached 70% confluence. BeWo cells were incubated either with medium containing 0.45% DMSO or 30µM FSK/0.45% DMSO for 48 h, medium was removed and stored at -20 °C until the β-hCG, progesterone and estradiol concentration was determined in the central laboratory of the Universitätsklinikum des Saarlandes, Germany via electrochemiluminescence immunoassay (ECLIA).

### BeWo cells cultured with low calcium medium

For each experiment, cells were grown in four small cell culture flasks (Falkon, 25cm^2^) until confluence reached 70%. The medium was replaced either by the cell culture medium containing 0.94mM Ca^2+^ (Ca^2+^ norm/N), which corresponds to the general Ca^2+^ concentration used for the cultivation of BeWo cells, or reduced 0.35mM Ca^2+^ (Ca^2+^ low/L) with 30 μm FSK/0.45% DMSO (FSK) or with 0.45% DMSO (ctrl). The amount of EDTA was calculated with the Webmaxcstandard7/3/2009 software (0.6 mM EDTA) and calcium concentrations were validated using a Dri-Chem NX500i 546 System (FujiFilm, Japan) measurement. Cells were incubated for 48 h, and the cell culture supernatant was removed, and hormone concentration (β-hCG, progesterone and estradiol) was determined. The cells were harvested with a cell scraper (Corning, Tewksbury, MA, USA), centrifuged, washed with PBS, centrifuged again and the cell pellet was stored at -80 °C.

### Preparation of BeWo cell lysates for western blot proteome analysis

BeWo cell lysates were prepared from the frozen cell pellets. Pellets were resuspended with 2 ml RIPA lysis buffer (150mM NaCl, 50mM Tris HCl pH 8, 5mM EDTA, 1% Nonidet P40, 0.1% SDS, 0.5% Na-deoxycholate + protease inhibitors cocktail (Roche, Mannheim, Germany) and the cells were lysed using ultrasound sonification (Bandelin, Berlin, Germany). Cell suspension was sheared 10 times (24G gauge needle) and incubated for 30 min at 4 °C on a shaker. After incubation, the suspension was centrifuged using an ultracentrifuge (Beckmann Coulter Optima^™^ MAX-E ultracentrifuge) at 100.000xg at 6 °C for 1 h. The supernatant containing the solubilized proteins was collected and was used for further experiments.

### Protein concentration determination and precipitation with trichloroacetic acid (TCA)

The protein amount was determined using the Pierce^™^ BCA Protein Assay Kit (ThermoFisher, Waltham, MA, USA). Due to the low sample concentrations, for MS analysis 200 µg protein in the lysate was mixed with the same volume of 40% TCA stock solution (4 °C), incubated on ice for 20 min, centrifuged at 2 °C for 10 min at 14,000 rpm and the supernatant was discarded. The pellet was washed twice with two volumes of acetone, mixed, centrifuged and the supernatant was discarded. The pellet was dried at room temperature in an open tube and reconstituted in 30 µl 2x denaturing buffer (SDS 8% (w/v) TRIS pH 6.8 120mM, bromophenol blue 0.01% (w/v), glycine 20% (v/v), β-mercaptoethanol 10% (v/v)).

### Western blot

30 µg of protein lysates (1:1 diluted with 2x denaturing buffer) were denatured at 60 °C for 20 min and separated in a 4–12% SDS-polyacrylamide Bolt Bis-Tris gradient gel (Invitrogen, Schwerte, Germany) using MOPS running buffer (50 mM MOPS, 50 mM Tris, 0.1% SDS, 1 mM EDTA, pH 7.7). After separation, the proteins were transferred to nitrocellulose membrane (Trans-Blot Turbo Transfer System, Bio-Rad Laboratories, Feldkirchen, Germany), blocked and incubated with antibodies as described before [24]. Aromatase antibody from mouse (1:100, sc-374176, Santa Cruz Biotechnology, USA) or HSD11B2 antibody from rabbit (1:200, sc-20176, Santa Cruz Biotechnology, USA) was incubated overnight at 4 °C and the secondary anti-mouse HRP antibody (1:10 000, NA9310V, GE Healthcare UK Limited, Amersham) or anti-rabbit HRP antibody (1: 30 000, NA9340V, Cytiva, Buckinghamshire, UK) for 1 h at room temperature. Calnexin antibody (1:1000, SPA865, Enzo Life Sciences, Lörrach, Germany) incubation was used as loading control. The densitometric analysis was performed with the AIDA Image Analysis Software.

### Preparation of BeWo cell lysates for proteome analysis

200 µg of the concentrated BeWo cell lysates were loaded on a 4–12% gradient gel (Bolt, ThermoFisher, Karlsruhe, Germany) and separated by gel electrophoresis using electrophoresis buffer. The proteins were fixed on the gel with a 40% ethanol- and 10% acetic acid solution, the gel was washed with demineralized water and the proteins were visualized with a colloidal Coomassie staining solution (0.12% (w/v) Coomassie G250, 10% (w/v) ammonium sulphate, 10% (v/v) phosphoric acid, 20% (v/v) methanol in H_2_0). Eight bands per sample were cut and alternately washed with wash solution A (50 mM NH_4_HCO_3_) and B (50 mM NH_4_HCO_3_ and 50% (v/v) acetonitrile). Disulfide bridges were reduced with 10mM dithiothreitol (in wash solution A) for 30 min at 56 °C, the free thiol groups were carbamidomethylated with 5 mM iodoacteamide (in wash solution A) incubation in the dark, and the gel bands were washed alternately with wash solutions A and B again. After drying the bands in a vacuum centrifuge, they were incubated overnight with 15 µl porcine trypsin (10 ng/µl, Promega) at 37 °C. Tryptic peptides were extracted twice after incubation with an extraction buffer (2.5% formic acid, 50% acetonitrile) in an ultrasonic bath. Combined extracts were dried in a vacuum centrifuge and resuspended in 19 µl 0.1% (v/v) formic acid.

### Proteome analysis with Nano-LC-HR-MS/MS

Label free proteome analysis was done four times (two biological and technical replicates) for each of the four different cell culture conditions: (1) Ca^2+^ norm (N) DMSO (ctrl), (2) Ca^2+^ norm (N) FSK, (3) Ca^2+^ low (L) DMSO (ctrl) and (4) Ca^2+^ low (L) FSK, respectively. In total 144 fractions were analyzed by LC-ESI-HRMS/MS as described at Diener et al. 2023 [25] with minor modifications. Tryptic peptides were analyzed by data-dependent nano-LC-ESI-HR-MS/MS analysis using the instrumentation setup: Ultimate 3000 RSLC nano system, Ultimate3000 RS autosampler, Nanospray Flex Ion source coupled to a Thermo Scientific Orbitrap Eclipse Tribrid mass spectrometer (Thermo Scientific, Germany). Peptides were separated with a gradient generated with buffer A (water and 0.1% formic acid) and buffer B (90% acetonitrile and 0.1% formic acid) at a flow rate of 300 nl/min: 0–5 min 4% B, 5–80 min to 31% B, 80–95 min to 50% B, 95–100 min to 90% B, 100–105 min hold 90% B, 105–106 min to 4% B and 106–120 min to 4% B. Peptides were trapped on a C18 trap column (75 μm × 2 cm, Acclaim PepMap100C18, 3 μm) and separated on a reverse phase column (nano viper DNV Pep Map^™^ Neo capillary column, C18; 2 μm; 75 μm × 50 cm). The chromatography effluent was sprayed into the mass spectrometer using a nano ESI emitter (stainless steel, Thermo Scientific, ionization energy: 2.4 keV). MS1 peptide spectra were acquired using the Orbitrap analyzer (*R* = 120k, RF lens = 30%, m/z = 375–1500, MaxIT: auto, profile data, intensity threshold of 10^4^). Dynamic exclusion of the 10 most abundant peptides was performed for 60 s. MS2 spectra were collected in the linear ion trap (isolation mode: quadrupole, isolation window: 1.2, activation: HCD, HCD collision energy: 30%, scan rate: fast, data type: centroid).

### Raw LC-MS data analysis

Raw file analysis was performed with Proteome Discoverer 3.0 (Thermo Fisher Scientific) using the Sequest HT algorithm to search against reviewed UniProt Homo sapiens database (20 354 sequences, Version November 2023). The raw files of the individual bands belonging to a sample were combined as fractions of the sample. The database search parameters were used as follows: trypsin as full proteolytic enzyme, peptides with up to two missed cleavage sites, precursor mass tolerance of 10ppm for finding peptide candidates, fragment mass tolerance of 0.6 Da for matching fragment peaks, a minimum peptide length of 6 and a maximum length of 144 with max number of peptides reported of 10, Cystein carbamidomethylation as static and oxidation (M) and N-acetylation as dynamic modification. The Peptide Spectrum Matches (PSM) validation with “Percolator” occurred using “Target/Decoy strategy” whereby validation based on q-value and false discovery rate (FDR) cutoff filter was set to 0.01. As part of label free quantification (LFQ) “Minora Feature Detector” was used to detect chromatographic peaks in MS1 data and charting them to peptide spectral matches. Just high confident PSM were used for component verification. Another part of quantification is to map features from different raw files by retention time alignment using “Feature mapper” node with a maximum retention time shift of 10 and a max mass tolerance of 10ppm allowed. Modifications with a lower site-probability threshold than 75/100 were not shown for peptides (“PSM Grouper”). The “Precursor Ion Quantifier” was main component of LFQ whereby precursor abundances based on intensity. Each protein group contained at least two peptides and in one of four comparisons at least 2 unique peptides were included for quantification. All peptides were used that were not shared between different proteins or protein groups and shared peptides were assigned to protein that had more identifications but not for the other proteins they were contained in. To correct for experimental bias the program used the total peptide amount for normalization. Protein abundances were calculated by summing sample abundances of the connected peptide groups. Proteome discoverer software used pairwise ratio method for fold change analysis and identification of differences. The abundance ratios were calculated as median of all possible pairwise ratios calculated between replicates of all connected peptides with a maximum fold change of 100. No missing value imputation was done. PD3.0 software used t-test based on distribution of “background” proteins for *p*-value calculation to complete statistical testing and visualized the results by generating volcano plot. The x-axis of the volcano blots displays the log2 fold change value and y-axis the log 10 *p*-value (*p*-value significance setting 0.05; -log10 (0.05) = 1,3).

### Bioinformatic analysis

Proteins, that were identified as significantly dysregulated were further analyzed by GO term enrichment analysis (for biological processes) with the free software ShinyGO 0.80 with 0.05 FDR cutoff (http://bioinformatics.sdstate.edu/go/) (Xejin G, Jianil Q, Spors E et al. South Dakota State University (SDSU)) and GOrilla (BMC Bioinformatics 2009, 10:48) (Eran Eden, Doron Lipson, Sivan Yogev, Zohar Yakhini). To illustrate biological variances between samples, heat maps were created with the program Morpheus (http://software.broadinstitute.org/morpheus) using normalized abundance values of each protein.

### Statistical analysis and figures

The statistical analysis of hormone levels, fusion index and densitometric analysis of western blot was performed with GraphPad Prism 8.0.2. When comparing two groups, the unpaired t-test was used. Multiple conditions were compared with a One-way ANOVA test corrected for multiple comparisons using Bonferroni test. A significance level of *p* < 0.05 was selected for both tests. Graphical Figures were created with BioRender.com and CorelDraw X7 Version 17.0.0.491.

## Results

In cell culture, FSK treatment was used to stimulate adenylate cyclase and thus increases the intracellular cAMP concentration. cAMP can in turn activate the cAMP-dependent protein kinase A (PKA) and cAMP response element binding protein (CREB) [26] and subsequently controls cellular mechanisms such as gene transcription. In BeWo cells, PKA activation ultimately leads to the fusion of the trophoblast-like cells [2]. Therefore, BeWo cells cultured with DMSO (negative control) represent cytotrophoblast-like cells and treatment with FSK induces differentiation in syncytiotrophoblast-like cells (STB-like cell). To validate possible changes in cell morphology as well as tight and adherend junctions of the trophoblast-like cells, the localization of the tight junctional protein zona occludens-1 (ZO-1) in the presence or absence of FSK was investigated by immunofluorescence (Suppl. Figure 1). The size of the BeWo epithelial cells and their cell nuclei increased after FSK treatment (Suppl. Figure 1 A). In contrast, the length of continuous ZO-1 staining between adjacent cells decreased, indicating the disruption of tight junctions resulting in the presence of multinucleated fused cells. Moreover, after FSK treatment BeWo cells show a high fusion index ((NNS-S)/T) x 100%, NNS = number of nuclei in syncytia, S = number of syncytia, T = total number of nuclei) (Suppl. Figure 1B). The β-hCG concentration also increased significantly after FSK (Suppl. Figure 1 C), whereby the stimulated BeWo cells in the cell culture secreted an average of 953 mIU/ml hCG.

### Syncytialization of cytotrophoblast-like cells depends on the extracellular calcium concentration

**Fig. 1:**
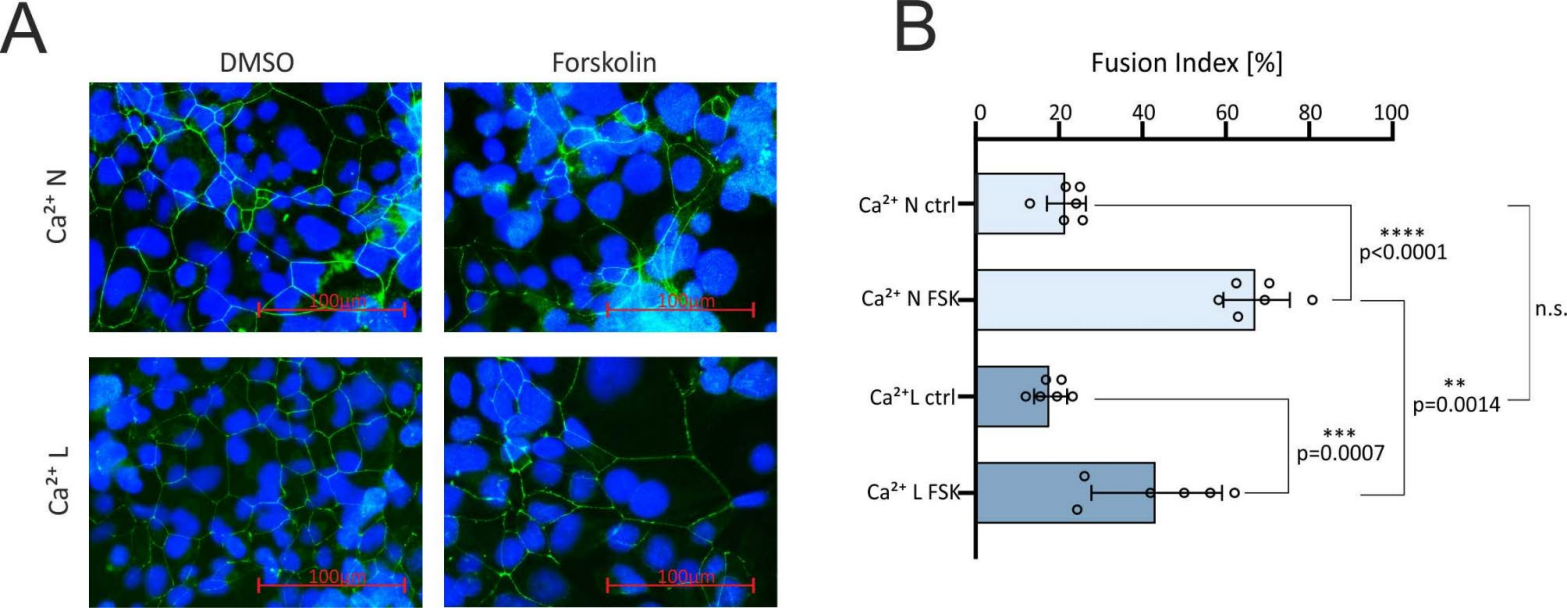
Calcium dependence of FSK induced syncytialization (**A**) ZO-1 immunostaining before (ctrl) and after 30µM FSK stimulation and under low (Ca^2+^ L = 0.35 mM) and normal calcium (Ca^2+^*N* = 0.94mM) cultured BeWo cells. (**B**) Fusion index of the differently cultured cells. Statistical analysis: One way ANOVA with Bonferroni correction, *N* = 6, *p* < 0.05

As shown in previous experiments, mouse trophoblasts and BeWo cell morphology and function depends on the presence of intra-and extracellular calcium [19, 21, 22]. BeWo cells cultured in the presence of different calcium concentrations (Suppl. Figure 2) show an altered morphology at 0.35mM Ca^2+^ but are still able to grow. Therefore, we decided to compare the normal Ca^2+^ concentration in the medium of 0.94 mM, with the 0.35 mM concentration, which was chosen as the low-calcium condition. First, the influence of the reduced extracellular calcium concentration on cell fusion was visualized by immunostaining with the ZO-1 antibody. Therefore, the calcium concentration was reduced from 0.94 mM (in the following named as Ca^2+^ normal and labelled in figures as Ca^2+^ N) to 0.35mM (in the following named as Ca^2+^ low and labelled in figures as Ca^2+^ L) in the medium, BeWo cells were treated with FSK or DMSO (Fig. 1A) and the fusion index was determined. In the presence of FSK the fusion index was significantly higher under both low and normal calcium concentrations. The fusion index was significantly higher in the presence of FSK and normal calcium compared to cells cultured in low calcium medium and FSK (Fig. 1B), whereas the fusion index of cells cultured with DMSO was not significantly different. The experiment shows that the fusion of the trophoblasts also depends on the extracellular calcium concentration.

### β-hCG, estradiol and progesterone secretion is differentially stimulated by calcium

**Fig. 2:**
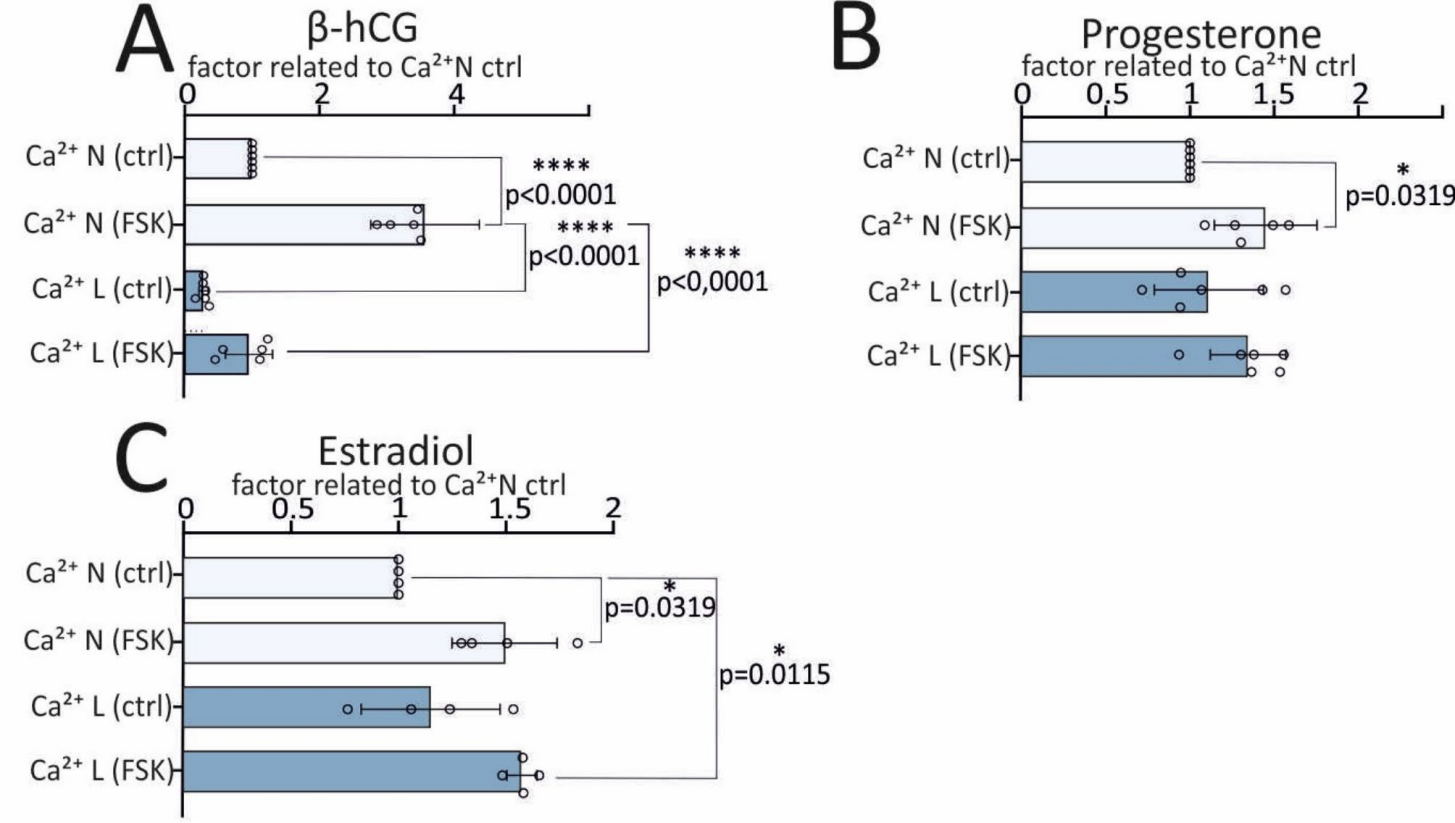
The hormone secretion of BeWo cells depends on FSK treatment and that of β-hCG also on the extracellular calcium concentration. **A-C**, cells were incubated in the presence of low (0.35mM) or normal (0.94mM) calcium and stimulated with 30µM FSK. Control cells were treated with DMSO. Hormones were determined in the supernatant: β-hCG (**A**), progesterone (**B**) and estradiol (**C**). Hormone levels were normalized to 1 µg protein. Hormone release in the presence of normal calcium without FSK stimulation was set to 100% (factor 1). Statistical analysis: One way ANOVA with Bonferroni correction, N (β-hCG and progesterone) = 6, N (estradiol) = 4

The pregnancy hormone and syncytialization marker β-hCG is involved in different processes in syncytiotrophoblasts [3, 6, 8, 9]. It is secreted autocrine and controls cytotrophoblast cell fusion and steroid hormone synthesis [27, 28]. We next tested if calcium has an impact on hCG secretion (Fig. 2A).

As expected, the β-hCG secretion increased after BeWo cell incubation with FSK in the presence of normal and reduced calcium exposure about 3.6 and 3.4 times, respectively (Suppl. Figure 1 C, Fig. 2A). However, the basal β-hCG secretion is lower in cells in the presence of low calcium (Suppl. Figure 3 C) as well as after induction of syncytialization with FSK (Fig. 2A, Suppl. Figure 3B). After syncytialization of BeWo cells under low calcium conditions, only 1/3 of the regular hCG level achieved under normal calcium conditions after FSK treatment could be measured in the supernatant. This difference was also observed without FSK treatment (Suppl. Figure 1 C, Fig. 2A). These results show that the β-hCG secretion of cytotrophoblast-like and the β-hCG secretion of STB-like cells are calcium dependent. The concentrations of progesterone (mean value: 56.6 ng/m) and estradiol (mean value: 901.8 pg/ml) also significantly increase after FSK treatment (Fig. 2B and C, Suppl. Figure 3D and G) in the presence of 0.94 mM calcium. However, compared with β-hCG, the secretion of estradiol and progesterone is not calcium dependent (Fig. 2B and C, Suppl. Figure 3 F, I).

### Proteome analysis of cytotrophoblast- and syncytiotrophoblast-like phenotypes

To monitor potential changes of protein expression during syncytialization we first analyzed multiple sets of proteome experiments using BeWo cells that were not stimulated (ctrl) or stimulated by FSK (48 h) to induce syncytialization. In both experiments, cells were cultured with 0.94 mM calcium (Ca^2+^ N). After incubation in the presence/absence of FSK, cell lysates were prepared, same protein amounts were separated on a denaturing gel, fractionated and tryptic digested. Tryptic peptides were extracted and analyzed by nano-LC-HR-MS/MS analysis. The different fractions belonging to one sample were combined and a qualitative and semi quantitative calculation with a label free quantification method based on precursor ion intensity (abundance value) was performed.

**Fig. 3:**
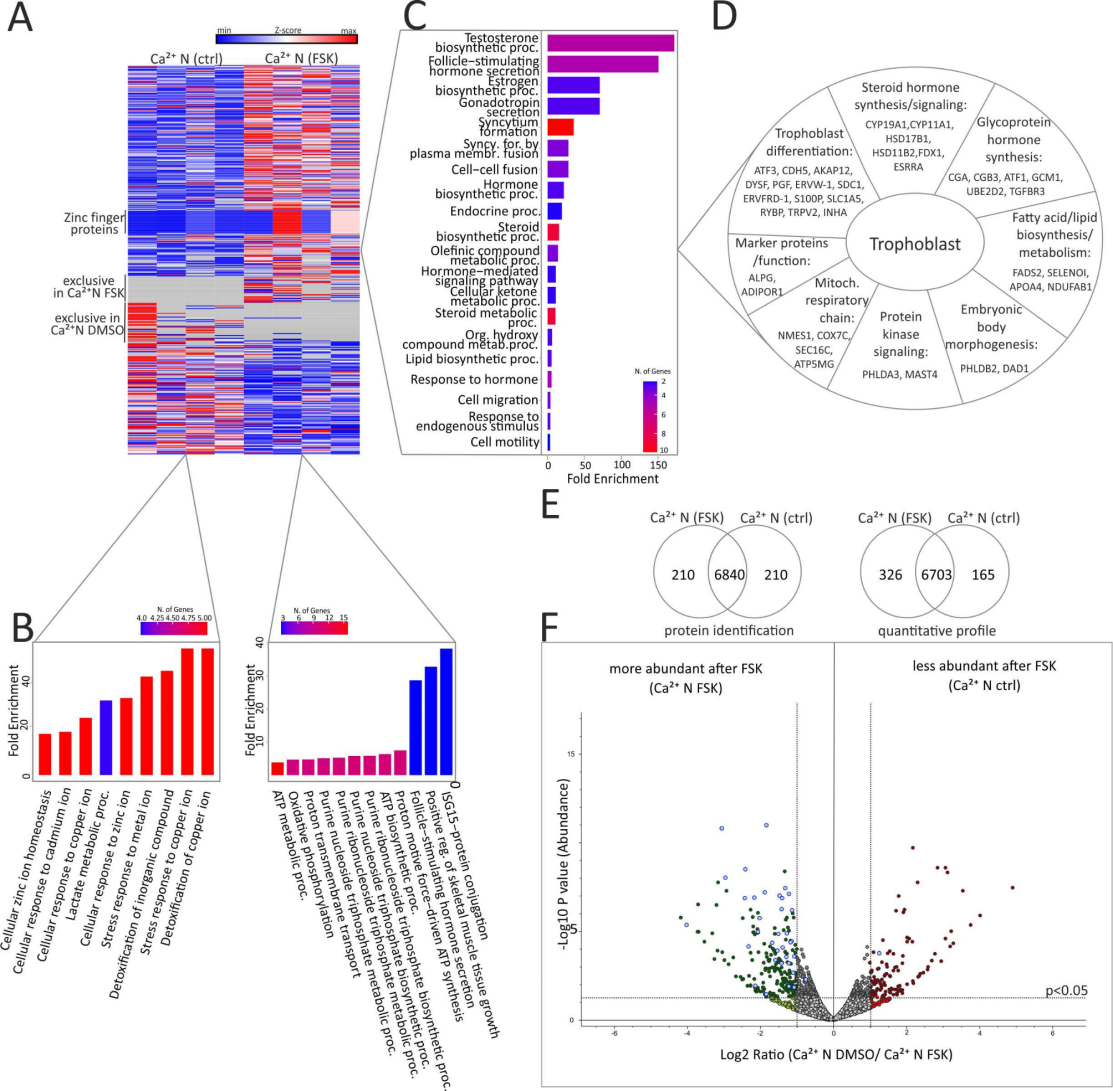
Proteome analysis of RIPA lysates from BeWo cells cultured under normal Ca^2+^ condition (0.94mM = Ca^2+^ N) and 48 h incubation with DMSO (ctrl) or with FSK (2 biological and 2 technical replicates). **(A)** Heatmap of all dysregulated proteins (491) during differentiation of cytotrophoblasts to STB. Normalized abundance value of each sample was used for quantification. **(B)** Increased signaling pathways before (left) and after (right) induction of syncytialization with FSK in cells cultured under normal Ca^2+^ conditions. **(C)** Gene ontology analysis (ShinyGO) to determine increased signaling pathways of selected dysregulated proteins due to placental expression (UniProt). **(D)** Differentially expressed proteins shown in C are grouped according to their functional categories of gene ontology. **(E)** Venn diagram of identified and quantified proteins before (Ca^2+^ N ctrl) and after (Ca^2+^ N FSK) FSK induced syncytialization. **(F)** Volcano plot of quantified proteins in DMSO (Ca^2+^ N ctrl) - and FSK treated cells (Ca^2+^ N FSK). *N* = 4, unpaired t-test. Blue point = proteins of D, red point = upregulated in Ca^2+^ N FSK, green points = upregulated in Ca^2+^ L FSK, grey point = no significant different expression

In BeWo trophoblast-like cells a total of 7260 proteins was identified before and after inducing syncytialization (Ca^2+^ N ctrl, *N* = 4 vs. Ca^2+^ N FSK, *N* = 4) (Fig. 3E, Suppl. Table 1). After cell treatment with FSK, 326 proteins were upregulated or exclusively expressed (**green spots** Fig. 3E and F, Suppl. Table 1), indicating that the expression of these proteins is induced during cytotrophoblast cell fusion and is characteristic for STB cellular maintenance. This group of proteins includes well-known markers for placental epithelial cells such as nectin-4 [29], cytokeratin-17 [30] and syndecan-1 [31], transcription factors (e.g. zinc finger proteins, GCM1), mitochondrial enzymes, candidates involved in protein kinase signaling or fatty acid and lipid biosynthesis. However, transporters and the transient receptor potential subfamily V member 2 (TRPV2) (Fig. 3A and D, Suppl. Figure 6), a calcium permeable ion channel which was previously mentioned at the transcriptome level as part of murine placenta development, were also upregulated after FSK cell treatment. Gene Ontology (GO) term analysis of the proteins upregulated after FSK stimulation reveals GO term enrichment for biological processes like ISG15-conjugation which was already mentioned in connection with pre-eclampsia [32], tissue growth, hormone secretion or ATP- synthesis (Fig. 3B **right**). On the other hand, 165 proteins occurred to be downregulated after syncytialization (Fig. 3E, **red spots** Fig. 3F, Suppl. Table 1). Most of them are related to detoxification, stress response or processes involved in Cu/Zn ion homeostasis (Fig. 3B left). Of the 491 deregulated proteins we detected in this study, 40 proteins (Fig. 3D, **blue spots in** Fig. 3F) have an affiliation to syncytialization processes, steroid and glycoprotein hormone metabolism, or lipid synthesis (Fig. 3C) or are listed as “tissue enriched (placenta)” in UniProt (Fig. 3D).

### Calcium dependence of the protein expression during syncytialization and hormone synthesis

**Fig. 4:**
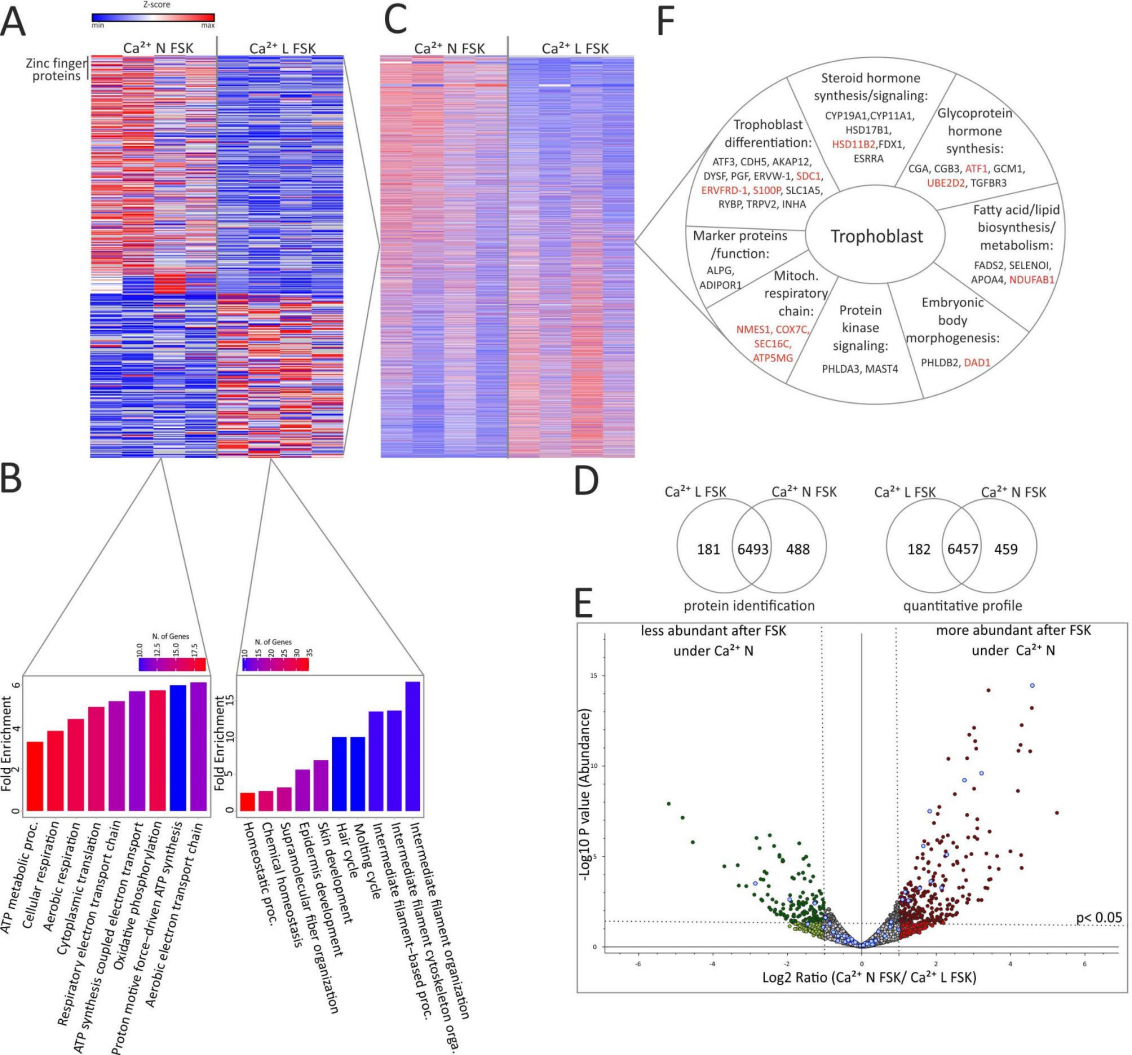
Proteome analysis of FSK stimulated BeWo cells cultured under normal (Ca^2+^ N) or under low calcium (Ca^2+^ L) conditions. **(A)** Heatmap of calcium dependent dysregulated proteins in FSK stimulated cells. **(B)** Increased signaling pathways in stimulated cells cultured under normal Ca^2+^ (left) and under low Ca^2+^ (right). Normalized abundance value of each sample was used for quantification. **(C)** Heatmap of all quantified proteins in stimulated BeWo cells. **(D)** Calcium dependent identification and quantification profile. **(E)** Volcano plot of quantified proteins in proteome analysis of stimulated BeWo cells. Identification of proteins that are upregulated by the activation of adenylyl cyclase (via FSK) in response to calcium (*p* < 0.05), *N* = 4, unpaired t-test. Blue point = Checked proteins from Fig. 4D, red point = upregulated in Ca^2+^ N FSK, green points = upregulated in Ca^2+^ L FSK, grey point = no significant different expression **(F)**. Differentially expressed proteins in differentiated BeWo cells shown in 4 C are grouped according to their functional categories of gene ontology. Proteins in red are up-regulated in FSK stimulated cells cultured under high calcium

Since we found that the changes in protein expression data matched with previously identified proteins with known syncytiotrophoblast function, and changes in calcium homeostasis seems to be related to some pregnancy associated pathologies, e.g. pre-eclampsia (PE), we checked whether and how protein expression profiles in cytotrophoblasts- and STB-like cells change in relation to a reduction of the extracellular calcium. For characterization of calcium dependence, the expression profiles of the following BeWo cell lysates were compared: (Ca^2+^ N ctrl) vs. (Ca^2+^ low ctrl) to determine calcium-dependent processes in cytotrophoblast-like cells, or (Ca^2+^ N FSK) vs. (Ca^2+^ low FSK) to determine calcium-dependent processes in STB-like cells, or (Ca^2+^ L ctrl) vs. (Ca^2+^ low FSK) to determine calcium-independent processes during syncytialization, whereby it is checked whether these proteins are also upregulated under regular calcium after syncytialization. (Fig. 4, Suppl. Figures 4, 5, Suppl. Tables 2, 3, 4).

Regarding the process of differentiation from BeWo cytotrophoblast- to STB-like cells (ctrl vs. FSK), calcium-independent syncytialization markers were identified that were significantly upregulated under both, reduced and normal calcium conditions after FSK stimulation (Ca^2+^ N ctrl vs. Ca^2+^ N FSK and in Ca^2+^ L ctrl vs. Ca^2+^ L FSK) (Fig. 4, Suppl. Figure 5, Suppl. Tables 1, 2, 4). This group includes the steroid hormone synthesis enzymes aromatase (CYP19A1) ) and the cholesterol-side chain cleavage enzyme (CYP11A1), the angiogenesis-stimulating placental growth factor (PGF), the junction protein cadherin-5 (CDH5) and dysferlin (DYSF) (Suppl. Figure 6), which has already been linked to cytotrophoblast fusion in model systems [10]. Moreover, the PKA regulator A-kinase anchor protein 12 (AKAP12), pleckstrin homology-like domain family A member 3 (PHLDA3), the transcriptional regulator RING1 and YY1-binding protein (RYBP), which has also been mentioned in connection with preeclampsia [33] and microtubule-associated serine/threonine protein kinase 4 (MAST4) were upregulated in both calcium conditions (Suppl. Tables 1, 2, 4, Suppl. Figure 6). Further syncytialization markers such as syncytin-1 GCM1, the steroid hormone synthesis enzyme 17-beta-hydroxysteroid dehydrogenase type 1 (HSD17B1) and the known preeclampsia marker inhibin alpha chain (INHA) as well as the calcium channel TRPV2 [20], the cadherin binding protein Cordon bleu protein like 1 (COBLL1) and Pleckstrin homology-like domain family B member 2 (PHLDB2) could not be clearly classified with regard to their calcium dependence (Suppl. Figure 6). They were only quantified as higher expressed by mass spectrometry under normal calcium (Ca^2+^ N ctrl vs. Ca^2+^ N FSK) conditions after FSK incubation (Suppl. Table 1) but there were no differences in experiments with calcium as variable factor (Suppl. Tables 2, 3, 4). In contrast to the previously mentioned proteins, however, numerous proteins were also formed in a calcium-dependent manner, for example determined by comparing the STB-like cell lysate either cultured with 0.94mM or 0.35mM calcium: 459 proteins were more abundant in FSK stimulated cells that were cultured under normal calcium (Fig. 4D, Suppl. Table 4) and 182 proteins were more abundant under low calcium. After syncytialization under normal calcium, proteins that are part of cellular respiration were mainly increased in expression (Fig. 4B, **left**), while pathway analysis (with ShinyGo) of upregulated proteins after syncytialization under low calcium generally revealed proteins that are part of biological processes such as fiber and organ development, intermediate filament associated processes and homeostatic processes (Fig. 4B, **right**). Regarding the general functions (identified with GOrilla) of proteins that were significantly higher expressed after syncytialization under normal calcium Ca^2+^N FSK, it is noticeable that 40 proteins with transcriptional regulatory activity (mainly zinc finger proteins, (Fig. 4A, Suppl. Table 2) and 21 proteins that are important for the structural constituent of ribosomes were identified. This observation is consistent with the functions of syncytiotrophoblasts, which are the main source of steroid and glycopeptide hormones in the placenta and are therefore considered to be a trigger of numerous pregnancy-preserving processes, most of which are driven by transcriptional induction [34–39]. In contrast, under reduced calcium (Ca^2+^ L FSK), proteins with zinc ion transmembrane transporter activity (SLC39A7, SLC39A9, SLC39A10), with sodium transmembrane transporter activity (SLC20A2, SLC38A2, SLC4A7, SLC23A2, SLC38A1) and with Ca^2+^ sensor activity (STIM2) were mainly upregulated, possibly for compensatory reasons.

Considering and comparing all cell culture conditions (Suppl. Tables 1–4) 22 of the 40 proteins (selected via UniProt for their known syncytiotrophoblast function/placental expression (**from** Fig. 3D, **blue spots in** Figs. 3E and 4E) were significantly deregulated in a calcium dependent manner. Table 5 (**supplement**) shows an overview of these 40 proteins (also seen in Figs. 3 and 4) and their expression levels in relation to the culture conditions. 13 proteins were significantly more expressed under regular calcium conditions in FSK stimulated BeWo cells (Suppl. Table 4). Of these proteins, some were detected as exclusively upregulated after FSK stimulation in the presence of regular calcium (NDUFAB1, S100P, SEC61G, NMES1, ATP5MG, DAD1, COX7) and some were also upregulated under reduced calcium. Interestingly their expression upregulation was higher (HSD11B2, SDC1, UBE2D2, ERVFDR-1) under regular calcium.

### The expression of enzymes for the synthesis of steroid hormones and the secretion of steroid hormones are increased during syncytialization

**Fig. 5:**
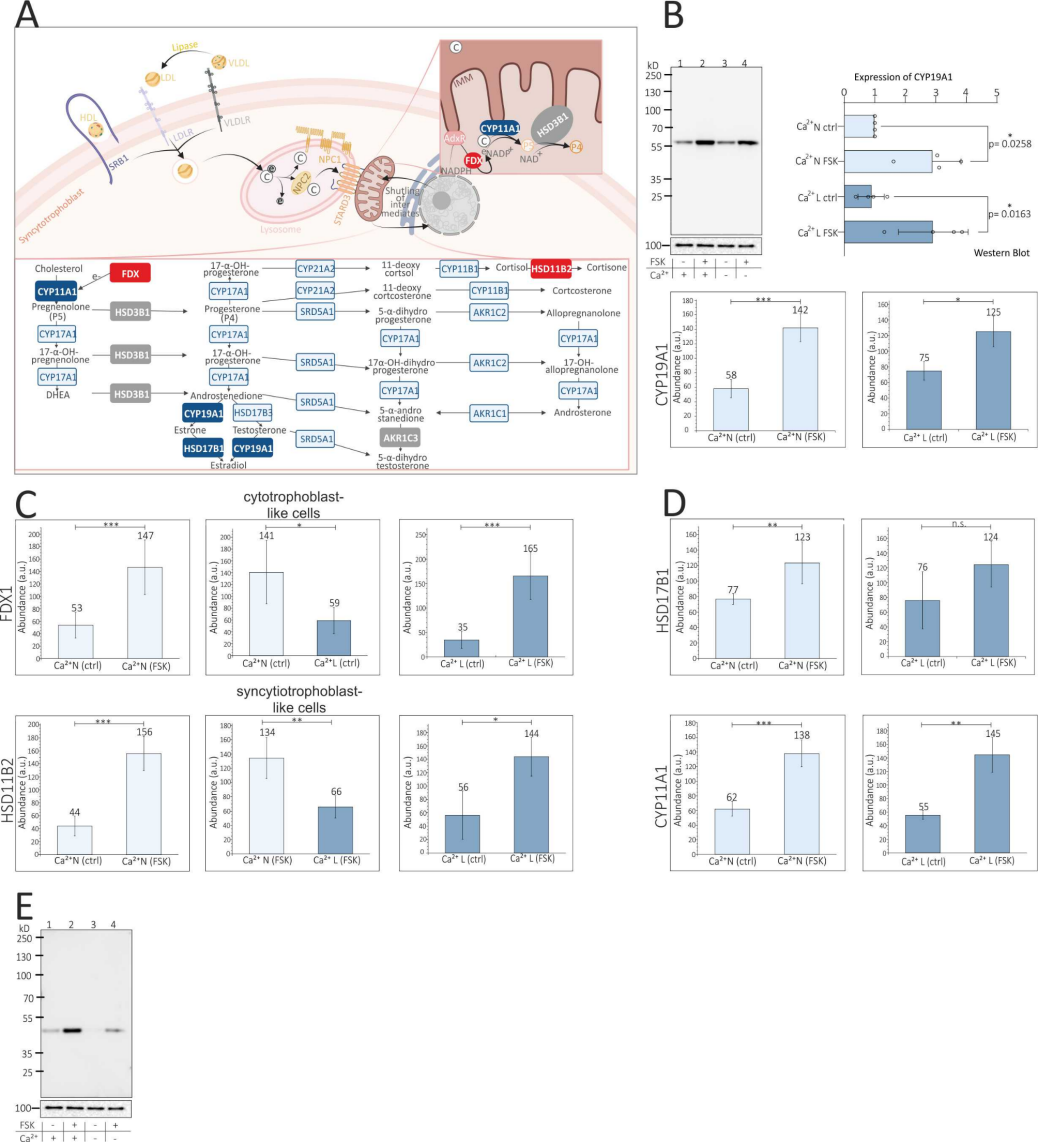
Model of proteins involved in steroid hormone synthesis, identified proteins in proteome of BeWo cells and calcium dependence of FDX and HSD11B2. **(A)** General schematic overview of steroidogenic pathways occurring in human tissues with enzymes involved in steroid hormone synthesis and potential intermediates. Mass spectrometrical identified proteins in BeWo cells are highlighted with filled boxes and white letters: in grey, proteins that were identified but not differentially expressed in a FSK or calcium-dependent manner, in blue, proteins that were upregulated in a calcium-independent manner after FSK stimulation and in red, proteins that were upregulated in a calcium-dependent manner after FSK stimulation in BeWo cells. **(B)** Expression analyzes of aromatase (CYP19A1). Top left, western blot of BeWo RIPA lysates incubated with antibodies directed to aromatase (top) and calnexin (bottom). Cells were cultured in the presence of normal calcium (lane 1), normal calcium + FSK (lane 2), low calcium (lane 3), low calcium + FSK. Top right, statistical analysis of 4 western blots normalized to calnexin. Aromatase amount, of cells grown in the presence of normal calcium was set to 100% (factor 1). One way ANOVA test, multiple comparison with Bonferroni, *p* < 0.05, *N* = 4. Bottoms left/right, MS analysis of aromatase (CYP19A1) abundance in BeWo cell lysates (*N* = 4) cultured in the presence of normal/low calcium +/- FSK, unpaired t-test, *N* = 4 **(C-D)** MS analysis of several dysregulated proteins involved in synthesis of steroid hormones in the presence of different calcium-concentrations (low L and normal N) +/- FSK, Adrenodoxin FDX (C, top), HSD11B2 (C, bottom), HSD17B1 (D, top) and CYP11A1 (D, bottom), *N* = 4, unpaired t-test **(E)** Western blot of lysates incubated with HSD11B2 antibody in the presence of normal calcium (lane 1), normal calcium + FSK (lane 2), low calcium (3), low calcium + FSK (lane 4) (E, top) and calnexin control (E, bottom)

Enzymes of steroid hormone synthesis were identified in the BeWo cell proteome (thickly marked in Fig. 5A) and, in addition, proteins that are upregulated in the presence of FSK were represented by bar charts (Fig. 5B-D). Aromatase, is the key enzyme for the synthesis of estradiol in trophoblasts. It is a cytochrome P450 monooxygenase that catalyzes the conversion of C19 androgens, androst-4-ene-3,17-dione (androstenedione) and testosterone to the C18 estrogens, estrone and estradiol, (Fig. 5A) [40, 41] and was detected during mass spectrometry and western blot (Fig. 5B).

Since the secretion of estradiol in the BeWo cells is increased during syncytialization but does not appear to be calcium-dependent (Fig. 2C), we also monitored the aromatase expression by western blot in BeWo cell lysates ( ≙ four culture conditions). Densiometric analysis of the stain intensity of the aromatase revealed that BeWo cells treated with FSK produced significantly more aromatase under both reduced and normal calcium conditions (Fig. 5B, **top**) and there is no significant relation to the calcium reduction, which was confirmed by mass spectrometric quantitative analysis (Fig. 5B, **bar charts below**, Suppl. Tables 1, 2, 4). Therefore, synthesis of aromatase shows no calcium dependency which is in line with the measured estradiol concentrations (Fig. 2C). However, aromatase expression itself also appears to be a syncytial marker. 17-beta-hydroxysteroid dehydrogenase type 1 (HSD17B1) also catalyzes the synthesis of estradiol from estrone (Fig. 5A). The expression of HSD17B1 was only significantly increased after FSK stimulation under normal calcium conditions (Fig. 5D, **top**, Suppl. Table 1, Suppl. Figure 6) (fusion marker) and shows the same tendency in the presence of reduced calcium. However, the HSD17B1-expression level of unstimulated (ctrl) and stimulated (FSK) cells was similar in the presence of low and high calcium. All these observations trend to favour calcium-independent estradiol synthesis in human STB.

Progesterone (P4) secretion revealed to be calcium independent (Fig. 2B). Cholesterol serves as the precursor for steroid hormone synthesis and is transported through the plasma membrane via lipoproteins [42] (Fig. 5A). The catalysis of cholesterol to progesterone takes place in the inner mitochondrial membrane. Several relevant actors are involved in this process: adrenodoxin reductase (AdxR), which transfers electrons from reduced NADP to the electron transfer protein adrenodoxin (FDX1), which provides the electrons for the catalysis reaction of cholesterol to pregnenolone by cholesterol-side chain cleavage enzyme P450 (CYP11A1). The resulting pregnenolone (P5) is converted to progesterone (P4) by 3 beta-hydroxysteroid dehydrogenase/Delta 5–>4-isomerase type 1 (HSD3B1) and cofactor NAD+ [43]. Except for adrenodoxin reductase, all proteins involved in the synthesis of progesterone were identified by proteome analysis. Here HSD3B1 expression showed no significant differences after either FSK stimulation or calcium reduction (Suppl. Tables 1–4), while CYP11A1 expression was found to be increased after FSK treatment, but showed no calcium dependence (Fig. 5D **bottom**, Suppl. Tables 1, 2, 4, Suppl. Figure 6). FDX1 is also a protein whose expression is induced by adenylyl cyclase activation during syncytialization. Regarding the FDX1 expression of FSK treated cells cultured under different calcium conditions the levels were comparable (Suppl. Table 4), but expression in unstimulated cells (ctrl) was significantly reduced when cells were cultured with low calcium (Fig. 5C, **top**, Suppl. Table 3, Suppl. Figure 6). Therefore, the expression of FDX1 may be impaired with reduced calcium in cytotrophoblasts. The expression of aldo-keto reductase family 1 member C3 (AKR1C3) also did not differ significantly after either FSK stimulation or calcium reduction (Suppl. Tables 1–4, Suppl. Figure 6). Contrary to that, the expression of 11-beta-hydroxysteroid dehydrogenase type 2 (HSD11B2), an enzyme involved in cortisol metabolism, showed calcium dependency in differentiated cells cultured in the presence of FSK (Fig. 5C **bottom**, 5E, Suppl. Table 4, Suppl. Figure 6), as determined by MS-analysis as well as visualized by Western blot.

## Discussion

Pregnancy associated pathologies like PE, IUGR or miscarriages may result from insufficient syncytialization of cytotrophoblast cells, defective synthesis of glucopeptide- or steroid hormones or inadequate feto-maternal exchange of nutrients like calcium [1, 6, 7]. The β-hCG level in the maternal blood increases exponentially by more than a hundredfold from the seventh day of conception (5-50mIU/mL) to the third week of pregnancy (18-7340mIU/mL) [1]. The syncytialization process of cytotrophoblasts is also accompanied by the production of β-hCG. Syncytiotrophoblasts are the main source of the pregnancy hormone and induce trophoblast invasion [44], syncytialization and synthesis of steroid hormones [45]. Compared to previous BeWo proteome studies [8, 46, 47], we were able to identify more than 7000 proteins and compare their expression profiles for the first time. The differences between the studies are due to different experimental parameters as well as different mass spectrometers. Interestingly, two studies found many proteins that we also identified and concluded that there is a high similarity between the proteomes of BeWo cells and those of placental tissue and villous trophoblasts [46, 47]. Our proteome analysis of cytotrophoblast- and STB-like cells showed, that the expression of many proteins, which are involved in syncytialization, depend on the presence of FSK in BeWo cells and some also on the extracellular calcium concentration, even if oxygen level and calcium concentrations were not fully equivalent to physiological conditions present in the placenta. Hereafter, we recapped the cellular processes taking place in STB and tried to categorize them regarding the relevance of calcium in cytotrophoblast- and STB-like BeWo cells.

β-hCG synthesis depends on cAMP mediated activation of kinases such as p38 [48] and PKA [49] and associated transcription factors. In addition, calcium activates PKC, which can also induce hCG synthesis [49]. The signaling of these protein kinases is regulated by anchor proteins (AKAPs) [50–53]. By comparing the different proteome datasets, we identified AKAP12 as a new syncytiotrophoblast fusion marker induced by adenylyl cyclase activation via FSK that was upregulated in a calcium-independent manner after syncytialization (Suppl. Tables 1, 2, 4, Suppl. Table 5, Suppl. Figure 6). The activated transcription factor ATF-1 was reported to be a relevant factor for embryo-maternal crosstalk [54]. We found that the expression of ATF1 in FSK treated cells depends on the extracellular calcium concentration (Suppl. Table 4, Suppl. Table 5, Suppl. Figure 6). The two subunits of hCG, the shared α-subunit (CGA) of the glycoproteins LH, FSH, TSH and hCG [55] and the placenta-specific beta-subunit, were both also significantly higher expressed in the presence of FSK and showed calcium dependence (Figs. 3 and 4, Suppl. Table 3, Suppl. Table 4, Suppl. Table 5, Suppl. Figure 6). The influence of calcium on hormone synthesis was also investigated in Leydig cells, where calcium induces testosterone synthesis via the protein kinase C signaling pathway [56] and in BeWo cells, where calcium promotes the secretion of β-hCG into the medium [57]. The fact that our study now revealed that several proteins involved in hCG synthesis are expressed in a calcium-dependent manner, as well as calcium-dependent hCG secretion (Fig. 2), at both cytotrophoblast- and STB-like cell level, confirms that calcium is not only important for fetal mineral supply, but also for the synthesis of the hCG hormone at the feto-maternal barrier.

**Fig. 6:**
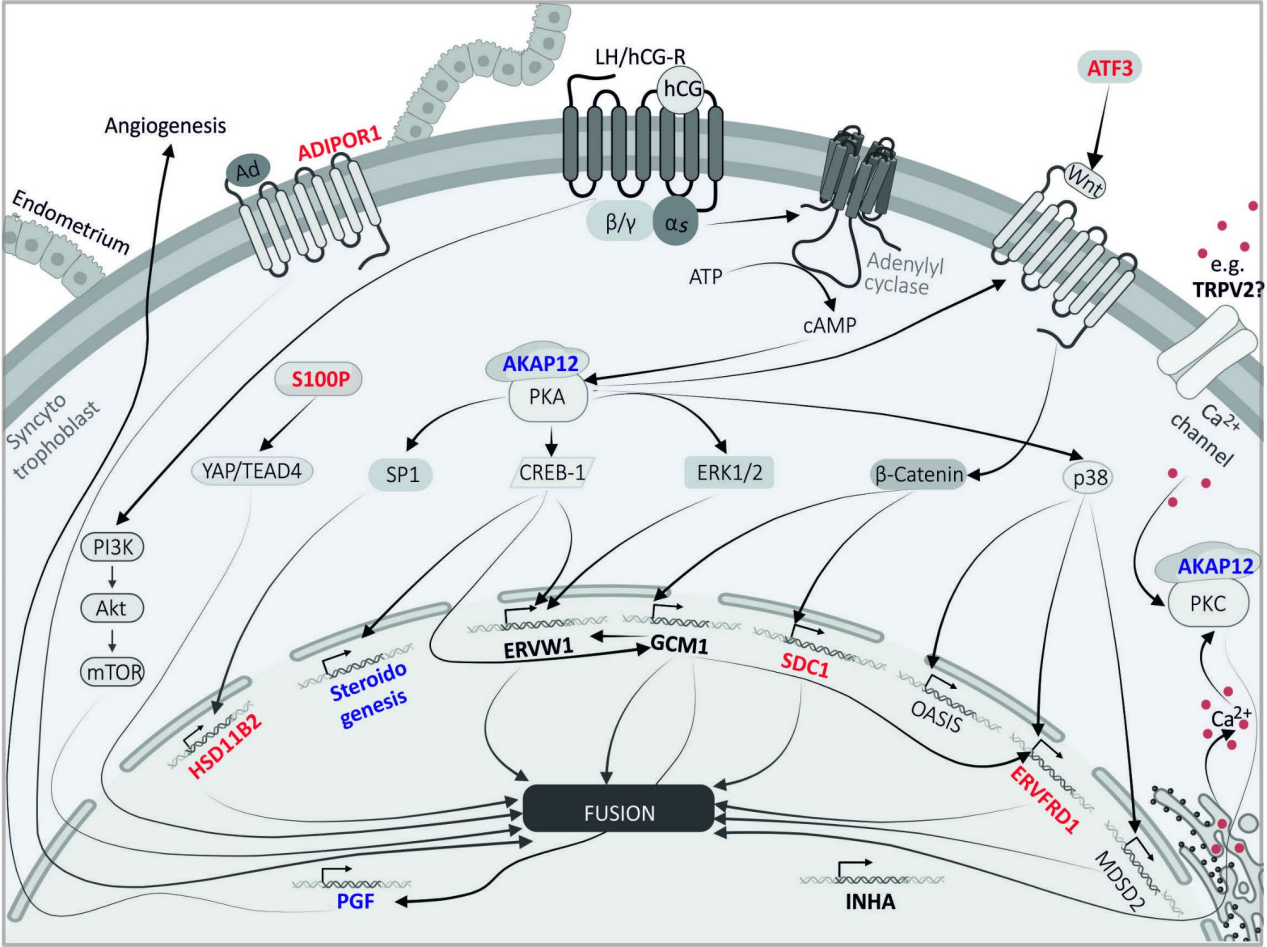
Model of possible signaling pathways that are partially stimulated by hCG and induce the synthesis of proteins with cell fusion-activating properties. Involved proteins identified in the proteomic analysis of BeWo cell lysates are shown in bold, with those classified as calcium-dependent in red and those classified as calcium-independent in blue. Activation of G_s_ protein-coupled protein receptors (e.g. LH/hCG) induces PI3K-AKT-mTor signaling pathway via the G_s_-beta-subunit [58], which in turn activates cell fusion [87] and receptor activation also induces adenylyl cyclase via the alpha subunit of the G_s_ protein. In this context, cAMP -PKA signaling can upregulate transcription factors SP1 [27] and CREB-1 [28] as well as the kinase ERK1/2 [28]. PKA can stimulate WNT ligands [88] and the p38 kinase [48] as well. SP1 can induce synthesis of HSD11B2, and CREB-1 can activate the synthesis of GCM1 transcripts [89], steroid genesis [28] and the synthesis of syncytin-1 [90]. The synthesis of GCM1 can also be induced by the WNT-β-catenin signaling pathway [71], which can also activate the synthesis of SDC1 [1]. P38-induced OASIS synthesis [48, 71] can also increase GCM1 synthesis. In turn, GCM1 induces PGF synthesis [72] and that of syncytin-2 (ERVFRD1), whose synthesis and that of its receptor MFSD2A can also be activated by p38 [48]. Transcript synthesis of syncytin-1 is also induced by ERK1/2 [1] and by GCM1. ATF3 may also be part of the Wnt signaling pathway in trophoblasts via miR-27 a-3p [91, 92]. S100P-activated YAP1/TEAD4 signaling pathways can also be part of placental development [79], as can adiponectin-ADIPOR-1-activated signaling pathways, and calcium itself can also act via the activation of PKC and thus induce trophoblastic cell fusion [71]

β-hCG is in turn secreted autocrinally by the STB and acts via the G_s_ protein-coupled lutropin-choriogonadotropic hormone receptor, which can induce numerous signaling cascades via AC-cAMP-PKA activation [27, 28] or by direct activation via the beta/gamma subunit (Pl3K-AKT-mTor signaling pathway [58],. This induces the transcription of various hCG-mediated proteins that are important for trophoblast function summarized in Fig. 6, including enzymes necessary for steroid hormone synthesis. Steroid hormone synthesis induced by FSK treatment has already been investigated in various cell lines and tissues, whereby the forskolin induced increase of progesterone and estradiol was already observed [59, 60] and was confirmed in our study (Fig. 2B, C) but no calcium dependence was observed in contrast to previous EGTA studies in placental cells [61–63]. Most of the studies focused on the influence of forskolin on steroid hormone synthesis in H29R cells, an endocrine cell line derived from human adrenocortical carcinoma. Here, FSK increased the expression of aromatase (CYP19A1), CYP17, CYP11A1 and CYP11B1 determined by RT-PCR [64–66] and corresponding products of steroid hormone syntheses such as estradiol and cortisol were also measured in a higher extent [67]. In addition, the incubation of MA-10 Leydig cells with FSK led to an increase of intracellular calcium, which was associated with higher levels of the steroid hormone progesterone [68]. Moreover, a calcium dependence at early steps of steroidogenesis has also been demonstrated by numerous independent observations in bovine glomerulosa cells [69]. However, regarding the influence of calcium on steroid hormone synthesis in BeWo cells, the proteome study and western blots (Figs. 4F and 5B-E, Suppl. Tables 1–4, Suppl. Figure 6) demonstrate that the expression of aromatase (CYP19A1), CYP11A1 and HSD17B1 is not calcium dependent, whereas the expression of FDX and HSD11B2 is. HSD11B2 is an enzyme of cortisol metabolism that converts bioactive cortisol into inactive cortisone and represents the feto-maternal glucocorticoid barrier [27] and was determined for the first time in our study to be expressed in a calcium-dependent manner in STB-like cells. It has already been mentioned to be upregulated by hCG during syncytialization to protect the fetus from excessive amounts of maternal glucocorticoids, as these are associated with pregnancy pathologies such as IUGR or the development of chronic diseases later in life [27]. The increase of cortisol may cause delayed fetal growth as consequence of bone resorption and mineral loss as it is the case in Cushing-syndrom patients [70].

In addition, we identified several proteins which were mentioned to be important in syncytiotrophoblast function (Fig. 6, gene names in bold). For example, syncytin-1 and the syncytin/PGF-activator GCM1 [48, 71, 72] are upregulated after FSK treatment which is in line with former reports [1–5, 7] (Suppl. Table 1, Suppl. Figure 6). Also, INHA and the cation channel TRPV2, which has been reported to be involved in the regulation of the placentation process in mice [20] are upregulated in FSK treated cells (Suppl. Table 1, Suppl. Figure 6). However, other TRP channel proteins, like TRPV6, showed low expression and were only detectable after enrichment by immunoprecipitation from the BeWo lysates [22]. The angiogenesis activator PGF and cell adhesion and syncytial marker protein CDH5 were upregulated under both normal and reduced calcium after syncytialization and represent calcium-independent markers of cell differentiation (Suppl. Tables 1, 2, Suppl. Figure 6).

In contrast, the receptor of the syncytin-1 protein SLC1A5 (Suppl. Tables 2, 3, Suppl. Figure 6) and the fusion protein syncytin-2 (ERVFRD-1) were found to be calcium-dependent. One of the functions of the p38 protein kinase is the induction of syncytin-2 synthesis [48], which triggers cell-cell fusion via its receptor MFSD2A [73]. The induction of syncytin-2 expression during syncytialization was also shown by our analysis. (Fig. 4D, F) and, additionally a calcium dependence of the achievable expression level after FSK induced syncytialization was determined (Fig. 4E, F, Suppl. Table 4). The higher expression after adenylyl cyclase activation (FSK) and calcium dependence was also observed for the ubiquitin conjugated enzyme E2 D2 (UBE2D2) (Suppl. Tables 1, 2, 4, Suppl. Figure 6) which, in addition to its ubiquitous functions as a ligase in various cells, was mentioned in the context of GCM1 stability. It is involved in degradation and ubiquitination of GCM1, and its knockdown led to a prolonged GCM1 half-life in vivo [74]. Increased expression after syncytialization with calcium dependence of the expression level was also observed for syndecan-1 (SDC1) (Fig. 4E, F, Suppl. Tables 1, 2, 4, Suppl. Figure 6). Dysregulations in the expression of proteoglycans such as SDC1 have been associated with fetal growth restriction and PE based on transcriptome data [75–78] and also serve as a prognostic marker for unfavorable pregnancy outcomes. The protein S100P, is one of the proteins that is exclusively expressed after FSK in the presence of normal calcium (Fig. 4E, F, Suppl. Table 4, Suppl. Figure 6), and is one of the factors that play an essential role in the regulation of trophoblast syncytialization [79, 80]. In JAR cells, another choriocarcinoma cell line, the upregulation of S100P correlated with the cell proliferation. Moreover, loss of S100P impaired ß-hCG secretion in the human cytotrophoblast stem cell model [79], and the expression of S100P was significantly reduced in placentas of patients with spontaneous abortions [80]. Next the potential compensatory upregulation of cyclic AMP-dependent transcription factor (ATF-3) expression during cell differentiation under reduced calcium (Suppl. Table 2) also implies that calcium participates in ATF-3 synthesis (Suppl. Figure 5). ATF-3 expression promotes trophoblast proliferation and has been reported to reduce inflammation in human fetal membranes [81]. ATF-3 deficiency is associated with recurrent implantation failure [82] and is part of PE pathology [83]. Moreover, the Transforming growth factor beta receptor type 3 (TGFBR3), which has been described as an Inhibin-coreceptor, is involved in the regulation of the FSH level in gonadotropic cells and affecting female fertility [84, 85], showed a high calcium dependency (Fig. 4E, F; Suppl. Tables 1–4, Suppl. Figure 6). Calcium had an expression-suppressing effect with and without FSK treatment. Finally, the adiponectin receptor protein 1 (ADIPOR1) was also classified as a calcium-dependent syncytial marker that is expressed significantly more under reduced calcium in both, undifferentiated and FSK stimulated BeWo cells (Fig. 3D, F; Suppl. Figures 4 and 6; Fig. 4E, F, Suppl. Tables 3, 4). This receptor, together with its ligand adiponectin, has also already been associated with the formation of the placental syncytium [6, 44, 86]. In earlier studies, the influence of calcium during syncytialization was investigated using GFP and dsRed-BeWo cell lines, for example, whereby syncytialization was quantified using fluorescence-activated cell sorting [4]. Here, an increase in intracellular calcium levels or nifedipine treatment reduced the extent of cell fusion. In our study, we decided to quantify snycytialization using ZO-1 staining, hormone assays and additionally by identifying numerous fusion markers using high-resolution mass spectrometry, and to reduce calcium concentration extracellularly. Considering the results of ZO-1 visualization and the calcium-dependent increase of the fusion index (Fig. 1) as well as the identification of various calcium-dependently expressed fusion markers we come to the conclusion that in addition to β-hCG synthesis and secretion, the syncytialization of trophoblasts seems to be also calcium- dependent.

## Conclusion

Taken together, in addition to confirming known syncytialization markers at the proteome level, numerous new proteins were identified for the first time in this study. Their expression is induced as part of the syncytial process. These include proteins involved in β-hCG and steroid hormone synthesis, suggesting that syncytialization and enzymes involved in steroid hormone synthesis are tightly linked. The expression of some of these proteins show a calcium dependency. In STB-like cells, cation channels are expressed in a compensatory manner if calcium is reduced, whereas under regular calcium conditions, transcription factors and components of the translation process are upregulated to a greater extent. Calcium therefore acts as a regulator of hormone expression and secretion. It is already known that an adequate calcium supply is important for the development of the growing fetus on many levels. However, this study provides deeper insights into trophoblast functions and shows that β-hCG expression, secretion and the syncytialization in general are calcium-dependent in trophoblast-like cells.

## Electronic supplementary material

Below is the link to the electronic supplementary material.

**Supplementary Material 1: Supplement Table 1**: Proteome Discoverer 3.0 result export of BeWo cell lysates +/- FSK (4 replicates) cultured with normal calcium (0.94 mM). Overview of all identified proteins and of dysregulated proteins with corresponding experimental q-value, program specific peptide score, protein coverage, peptide count, peptide spectrum matches, unique peptides, amino acid count, molecular weight, sequence score, abundance ratios, (adjusted) abundance ratio p-values, abundance ratios variability, grouped abundances, sample specific abundances, normalized abundances, normalized abundances, samples in which the protein was found, protein groups.

**Supplementary Material 2: Supplement Table 2**: Proteome Discoverer 3.0 result export of BeWo cell lysates +/- FSK (4 replicates) cultured with low calcium (0.35 mM).

**Supplementary Material 3: Supplement Table 3**: Proteome Discoverer 3.0 result export of BeWo cell lysates (4 replicates) cultured with DMSO and either with low (0.35 mM) or high (0.94 mM) calcium.

**Supplementary Material 4: Supplement Table 4**: Proteome Discoverer 3.0 result export of BeWo cell lysates (4 replicates) cultured with FSK and either with low (0.35 mM) or high (0.94 mM) calcium.

**Supplementary Material 5: Supplement Table 5**: Overview of dysregulated proteins in four analyzes. To create the table, each selected protein was marked in all four analyzes of Proteome Discoverer 3.0, in which two conditions were compared with each other, and the corresponding protein *p*-value of the abundance ratio, which was calculated using the unpaired t test from Proteome Discoverer 3.0, was selected. If there was a significant difference in one of the four analyzes (*p*-value of the protein in one analysis < 0.05), this is shown in the table with an arrow. In analysis 1, protein expression in cytotrophoblast-like cells (T) was compared to expression in STB-like cells (STB) cultured under normal calcium, to determine syncytialization markers in general. In analysis 2, protein expression in T cultured under normal calcium conditions was compared to expression in T cultured under low calcium conditions to determine calcium-dependently expressed proteins in cytotrophoblast-like cells. In analysis 3, protein expression in STB cultured under normal calcium was compared to expression in STB cultured under low calcium to determine calcium-dependently expressed proteins in syncytiotrophoblast-like cells. In analysis 4, protein expression in cytotrophoblast-like cells (T) was compared with expression in STB-like cells (STB) cultured under low calcium, representing the syncytialization process occurring under low calcium, to determine whether the syncytialization markers identified in analysis 1 were also upregulated after incubation with low calcium. In the table, the red arrow pointing upwards indicates upregulation of the protein in the comparison condition (second condition) and the blue arrow pointing downwards indicates downregulation in the comparison condition. The red-colored proteins showed calcium dependence at STB level, the orange-colored proteins at cytotrophoblast level and the black-colored proteins showed no differences in analysis 2 and 3, indicating a rather calcium-independent expression.

**Supplementary Material 6: Supplement Fig. 1**: Confirmation of FSK induced syncytialization of BeWo cells. **(A)** Immunofluorescence staining of tight junctions zona occludens protein 1 (ZO-1) (green) to visualize BeWo cell outlines and DAPI (blue) to stain the nuclei. Cells were treated with 30 µM FSK for 48h to induce fusion into syncytiotrophoblast-like phenotypes (STB). Cell treatment with DMSO was used as a negative control (ctrl) (≙ cytotrophoblast cells) **(B)** Determination of trophoblastic fusion index as ((NNS-S)/T) x 100% (NNS = number of nuclei in syncytia, S = number of syncytia, T = total number of nuclei) to analyze the level of multinucleated cells before and after FSK treatment. Analysis with unpaired t-test, *N* = 6 **(C)** Measurement of β-hCG hormone level from cell culture supernatant after 48h incubation with either DMSO or FSK as a marker for syncytialization. The determined hormone concentration was initially related to 1µg protein and afterwards normalized to reference concentration (ctrl) (factor = 1) within the experiment. Analysis with unpaired t-test, *N* = 6.

**Supplementary Material 7: Supplement Fig. 2**: Influence of the calcium concentration in the cell culture medium (extracellular calcium) on the morphology and confluence of BeWo cells, stained with Haema-Quick Stain Set, after 48h incubation time. Scale bar corresponds to 100µM.

**Supplementary Material 8: Supplement Fig. 3**: Influence of calcium on steroid hormone secretion of BeWo cells in cell culture supernatant. Cells were stimulated with 30µM FSK. DMSO was used as negative control. The determined hormone concentration was initially related to 1µg protein and afterwards normalized to reference concentration (factor = 1) within the experiment. Ca^2+^ L (low) = 0.35 mM, Ca^2+^ N (normal) = 0.94mM (**A-C)** β-hCG secretion in different treated cells. Analyzed with unpaired t-test, *N* = 6 (**D-F**) Progesterone secretion in different treated cells. Analyzed with unpaired t-test, *N* = 6 (**G-I**) Estradiol secretion in different treated cells. Analyzed with unpaired t-test, *N* = 6. Comparison of hormone secretion of BeWo cells treated with low calcium levels and either FSK or DMSO (A, E, H), of BeWo cells treated with FSK and either 0.94mM or 0.35mM calcium (B), of of BeWo cells treated with DMSO and either 0.94mM or 0.35mM calcium (C, F, I) or of BeWo cells treated with 0.94mM calcium and either FSK or DMSO (D, G).

**Supplementary Material 9: Supplement Fig. 4**: Proteome analysis before syncytialization under normal and under low calcium conditions. **(A)** Identification and quantification profile of proteome analysis. **(B)** Volcano plot of quantified proteins in proteome analysis of cytotrophoblast-like cells, *N* = 4, unpaired t-test. **(C)** Heatmap of quantified proteins. Lower right side: Heat map of all quantified proteins in proteome analysis of RIPA lysates from unstimulated cytotrophoblast under normal and under low calcium conditions (*p* < 0.05 and *p* > 0.05). Upper right side: Heat map of all dysregulated proteins. Lower left side: Heat map of selected proteins due to placental expression and due to classification as part of syncytialization from proteome analysis of stimulated and unstimulated trophoblast-like cells under normal calcium conditions (Fig. 3).

**Supplementary Material 10: Supplement Fig. 5**: Proteome analysis of undifferentiated and syncytializated trophoblasts under low calcium conditions. **(A)** Identification and quantification profile of proteome analysis. **(B)** Volcano plot of quantified proteins in proteome analysis of unstimulated and stimulated trophoblasts, *N* = 4, unpaired t-test. **(C)** Heatmap of quantified proteins under low calcium conditions. Lower right side: Heat map of all quantified proteins in proteome analysis (*p* < 0.05 and *p* > 0.05). Upper right side: Heat map of all dysregulated proteins. Lower left side: Heat map of selected proteins due to placental expression and due to classification as part of syncytialization from proteome analysis of stimulated and unstimulated trophoblasts under normal calcium conditions (Fig. 3).

**Supplementary Material 11: Supplement Fig. 6**: Comparison of the expression of ADIPOR1, AKAP12, AKR1C3, ALPG, ALPP, APOA4, ATF1, ATF3, ATP5MG, CHD5, CGA, CGB, COBLL1, COX7C, CYP11A1, CYP19A1, DAD1, DYSF, ERVFRD-1, ERVW-1, ESRRA, FADS2, FDX1, GCM1, HSD3B1, HSD11B2, HSD17B1, INHA, MAST4, NDUFAB1, NMES1,PGF, PHLDA3, PHLDB2, RYBP, S100P, SDC1, SEC61G, SELENOI, SLC1A5, TGFBR3, TJP1, TRPV2 and UBE2D2 in lysates of different cultured cells, visualized by bar charts (unpaired t-test).

## Data Availability

The datasets generated during and/or analyzed during the current study are available in the ProteoXchange repository the dataset identifier PXD054348 and 10.6019/PXD054348.

## Abbreviations

ADIPOR1: Adiponectin receptor protein 1
AKAP12: A-kinase anchor protein 12
ALPG: Alkaline phosphatase, germ cell type
APOA4: Apolipoprotein A4
ATF-1: AMP-dependent transcription factor
ATF-3: Cyclic AMP-dependent transcription factor
ATP5MG: ATP synthase subunit g, mitochondrial
Ca^2+^ L: Calcium low
Ca^2+^ N: Calcium normal
cAMP: Cyclic adenosine monophosphate
CDH5: Cadherin 5
CGA: Glycoprotein hormones alpha chain
CGB3: Choriogonadotropin subunit beta 3
COBLL1: Cordon bleu protein like 1
COX7: Cytochrome c oxidase subunit 7, mitochondrial
CYP11A1: Cholesterol-side chain cleavage enzyme
CYP19A1: Aromatase
DAD1: Dolichyl-diphosphooligosaccharide-protein glykosyltransferase subunit DAD1
DMSO: Dimethyl sulfoxide
DYSF: Dysferlin
EDTA: Ethylenediaminetetraacetic acid
ERVFDR-1: Syncytin-2
ERVW-1: Syncytin-1
ESI: Electro spray ionization
FADS2: Acyl-CoA desaturase FADS2
FDR: False discovery rate
FDX1: Adrenodoxin, mitochondrial
FSK: Forskolin
FSH: Follicle stimulating hormone
GCM1: Glial Cell Missing 1/Chorion-specific transcription factor GCMa
HCD: Higher-energy collisional dissociation
hCG: Human chorionic gonadotropin
HR: Hight Resolution
HSD11B2: 11-beta-hydroxysteroid dehydrogenase type 2
HSD17B1: 17-beta-hydroxysteroid dehydrogenase type
HSD3B1: 3 beta-hydroxysteroid dehydrogenase/Delta 5–>4-isomerase type 1
INHA: Inhibin alpha chain
IUGR: Intrauterine growth restriction
LC: Liquid chromatography
LFQ: Lable free quantification
LH: Luteinizing hormone
MAST4: Microtubule-associated serine/threonine protein kinase 4
MFSD2A: Sodium-dependent lysophosphatidylcholine symporter 1
MS: Mass spectrometry
N: Number of experiments
NNS: Number of nuclei in syncytia
NS: Number of syncytia
n: Number of cells
NDUFAB1: Acyl carrier protein, mitochondrial
NMES1: Normal mucosa of esophagus-specific gene 1 protein
OASIS: Cyclic AMP-responsive element-binding protein 3-like protein 1
PE: Pre-eclampsia
PGF: Placental growth factor
PHLDA3: Pleckstrin homology-like domain family A member 3
PHLDB2: Pleckstrin homology-like domain family B member 2
PKA: Protein kinase A
PKC: Protein kinase C
PSM: Peptide spectrum matches
RYBP: Regulator RING1 and YY1-binding protein
S100P: Protein S100-P
SDC1: Syndecan 1
SDS: Sodium dodecyl sulfate
SEC61G: Protein transport protein Sect. 61 subunit gamma
SLC1A5: Neutral amino acid transporter B (0) SLC1A5
SLC20A2: Sodium-dependent phosphate transporter 2
SLC23A2: Solute carrier family 23 member 2
SLC38A1: Sodium-coupled neutral amino acid symporter 1
SLC38A2: Sodium-coupled neutral amino acid symporter 2
SLC39A10: Zinc transporter ZIP10
SLC39A7: Zinc transporter SLC39A7
SLC39A9: Zinc transporter ZIP9
SLC4A7: Sodium bicarbonate cotransporter 3
STB: Syncytiotrophoblasts
STIM2: Stromal interaction molecule 2
T: Total number of nuclei
TCA: Trichloroacetic acid
TGFBR3: Transforming growth factor beta receptor type 3
TRIS: Tris (hydroxymethyl)aminomethane
TRPM7: Transient receptor potential cation channel subfamily M member 7
TRPV2: Transient receptor potential cation channel subfamily V member 2
TRPV6: Transient receptor potential cation channel subfamily V member 6
TSH: Thyroid Stimulating Hormone
UBE2D2: E3 ubiquitin-protein ligase E3D
ZO1: Zona occludens protein 1
