# Supplementary material for: The proteomic landscape of trophoblasts unravels calcium-dependent syncytialization processes and beta-chorionic gonadotropin (ß-hCG) production
Source: Reprod Biol Endocrinol. 2025 Mar 4;23:33. doi: 10.1186/s12958-025-01362-7 (PMC11877844; doi:10.1186/s12958-025-01362-7)
Supplement: Supplementary file 5 — Supplementary Material 5: Supplement Table 5: Overview of dysregulated proteins in four analyzes. To create the table, each selected protein was marked in all four analyzes of Proteome Discoverer 3.0, in which two conditions were compared with each other, and the corresponding protein p-value of the abundance ratio, which was calculated using the unpaired t test from Proteome Discoverer 3.0, was selected. If there was a significant difference in one of the four analyzes (p-value of the protein in one analysis < 0.05), this is shown in the table with an arrow. In analysis 1, protein expression in cytotrophoblast-like cells (T) was compared to expression in STB-like cells (STB) cultured under normal calcium, to determine syncytialization markers in general. In analysis 2, protein expression in T cultured under normal calcium conditions was compared to expression in T cultured under low calcium conditions to determine calcium-dependently expressed proteins in cytotrophoblast-like cells. In analysis 3, protein expression in STB cultured under normal calcium was compared to expression in STB cultured under low calcium to determine calcium-dependently expressed proteins in syncytiotrophoblast-like cells. In analysis 4, protein expression in cytotrophoblast-like cells (T) was compared with expression in STB-like cells (STB) cultured under low calcium, representing the syncytialization process occurring under low calcium, to determine whether the syncytialization markers identified in analysis 1 were also upregulated after incubation with low calcium. In the table, the red arrow pointing upwards indicates upregulation of the protein in the comparison condition (second condition) and the blue arrow pointing downwards indicates downregulation in the comparison condition. The red-colored proteins showed calcium dependence at STB level, the orange-colored proteins at cytotrophoblast level and the black-colored proteins showed no differences in analysis 2 and 3, indicating a rat [file 12958_2025_1362_MOESM5_ESM.docx]

- No different expression

Down-regulated in comparison condition (second condition)

Up-regulated in comparison condition (second condition)

Trophoblast= T (-> DMSO incubation)

Syncytiotrophoblast= STB (-> Forskolin incubation)

Analysis **1**: protein expression in T vs STB cultured under normal Ca^2+^

Analysis **2**: protein expression in T cultured under normal vs. T cultured under low Ca^2+^

Analysis **3**: protein expression in STB cultured under normal vs STB cultured under low Ca^2+^

Analysis **4**: protein expression in T vs STB cultured under low Ca^2+^

| **Analysis/**Name | **1** | **2** | **3** | **4** | **Analysis/**  Name | **1** | **2** | **3** | **4** |
| --- | --- | --- | --- | --- | --- | --- | --- | --- | --- |
| CGA |  | - |  |  | ATF3 | - | - | - |  |
| CGB3 |  |  | - |  | CDH5 |  | - | - |  |
| ATF1 | - | - |  | - | AKAP12 |  | - | - |  |
| GCM1 |  | - | - | - | DYSF |  | - | - |  |
| UBE2D2 |  | - |  |  | PGF |  | - | - |  |
| TGFBR3 |  |  |  |  | ERVW-1 |  | - | - | - |
| FADS2 |  |  |  | - | SDC1 |  | - |  |  |
| SELENOI | - | - | - |  | ERVFRD-1 |  | - |  |  |
| APOA4 |  |  | - |  | S100P |  | - |  | - |
| NDUFAB1 |  | - |  | - | SLC1A5 | - |  | - |  |
| PHLDB2 |  | - | - | - | RYBP |  | - | - |  |
| DAD1 |  | - |  | - | TRPV2 |  | - | - | - |
| PHLDB3 |  | - | - | - | INHA |  | - | - | - |
| MAST4 |  | - | - |  | CYP19A1 |  | - | - |  |
| NMES1 |  | - |  | - | CYP11A1 |  | - | - |  |
| COX7C |  | - |  | - | HSD17B1 |  | - | - | - |
| SEC61G |  | - |  | - | HSD11B2 |  | - |  |  |
| ATP5MG |  | - |  | - | FDX1 |  |  | - |  |
| ALPG |  |  | - |  | ESRRA | - |  | - | - |
| ADIPOR1 |  |  |  | - | COBLL1 |  | - | - | - |

| **Analysis** | **1** | **2** | **3** | **4** |
| --- | --- | --- | --- | --- |
|  |  | - |  | - |

Syncytialisation marker that is overexpressed

exclusively in the presence of normal Ca^2+^

Ca^2+^ dependency on STB level

- red marked protein name

| **Analysis** | **1** | **2** | **3** | **4** |
| --- | --- | --- | --- | --- |
|  |  | - |  |  |

Syncytialisation marker that is overexpressed

in the presence of normal and low calcium, but a sign. different expression level under low calcium

| **Analysis** | **1** | **2** | **3** | **4** |
| --- | --- | --- | --- | --- |
|  |  |  | - |  |

Ca^2+^ dependency on T level

- orange marked protein name

Syncytialisation marker that is overexpressed

in the presence of normal and low calcium, but a sign. different expression on basal T level

| **Analysis** | **1** | **2** | **3** | **4** |
| --- | --- | --- | --- | --- |
|  |  | - | - |  |

Ca^2+^ independency on STB level

- black marked protein name

Syncytialisation marker that is overexpressed

in the presence of normal and low Ca^2+^
