# Supplementary figures and images for: The proteomic landscape of trophoblasts unravels calcium-dependent syncytialization processes and beta-chorionic gonadotropin (ß-hCG) production
Source: Reprod Biol Endocrinol. 2025 Mar 4;23:33. doi: 10.1186/s12958-025-01362-7 (PMC11877844; doi:10.1186/s12958-025-01362-7)

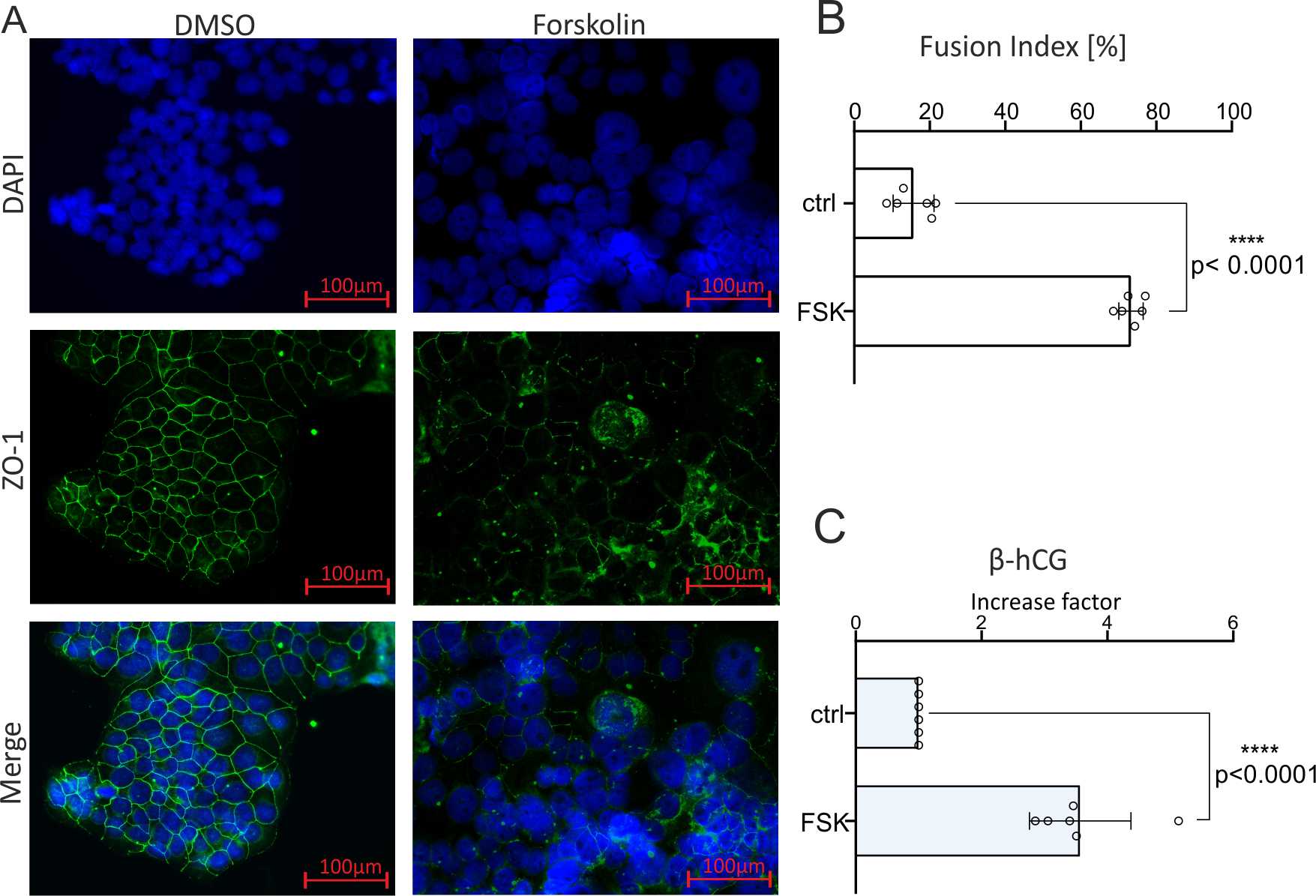

Supplement: Supplementary file 6 — Supplementary Material 6: Supplement Fig. 1: Confirmation of FSK induced syncytialization of BeWo cells. (A) Immunofluorescence staining of tight junctions zona occludens protein 1 (ZO-1) (green) to visualize BeWo cell outlines and DAPI (blue) to stain the nuclei. Cells were treated with 30 µM FSK for 48h to induce fusion into syncytiotrophoblast-like phenotypes (STB). Cell treatment with DMSO was used as a negative control (ctrl) (≙ cytotrophoblast cells) (B) Determination of trophoblastic fusion index as ((NNS-S)/T) x 100% (NNS = number of nuclei in syncytia, S = number of syncytia, T = total number of nuclei) to analyze the level of multinucleated cells before and after FSK treatment. Analysis with unpaired t-test, N = 6 (C) Measurement of β-hCG hormone level from cell culture supernatant after 48h incubation with either DMSO or FSK as a marker for syncytialization. The determined hormone concentration was initially related to 1µg protein and afterwards normalized to reference concentration (ctrl) (factor = 1) within the experiment. Analysis with unpaired t-test, N = 6. [file 12958_2025_1362_MOESM6_ESM.jpg]

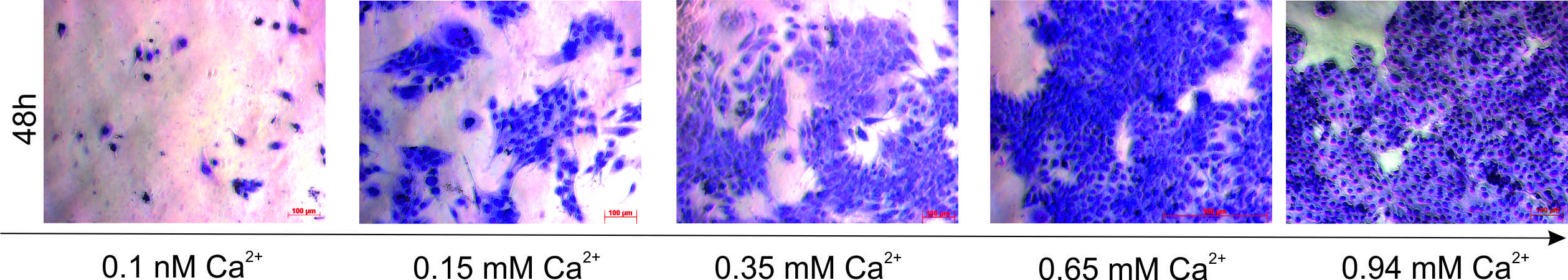

Supplement: Supplementary file 7 — Supplementary Material 7: Supplement Fig. 2: Influence of the calcium concentration in the cell culture medium (extracellular calcium) on the morphology and confluence of BeWo cells, stained with Haema-Quick Stain Set, after 48h incubation time. Scale bar corresponds to 100µM. [file 12958_2025_1362_MOESM7_ESM.jpg]

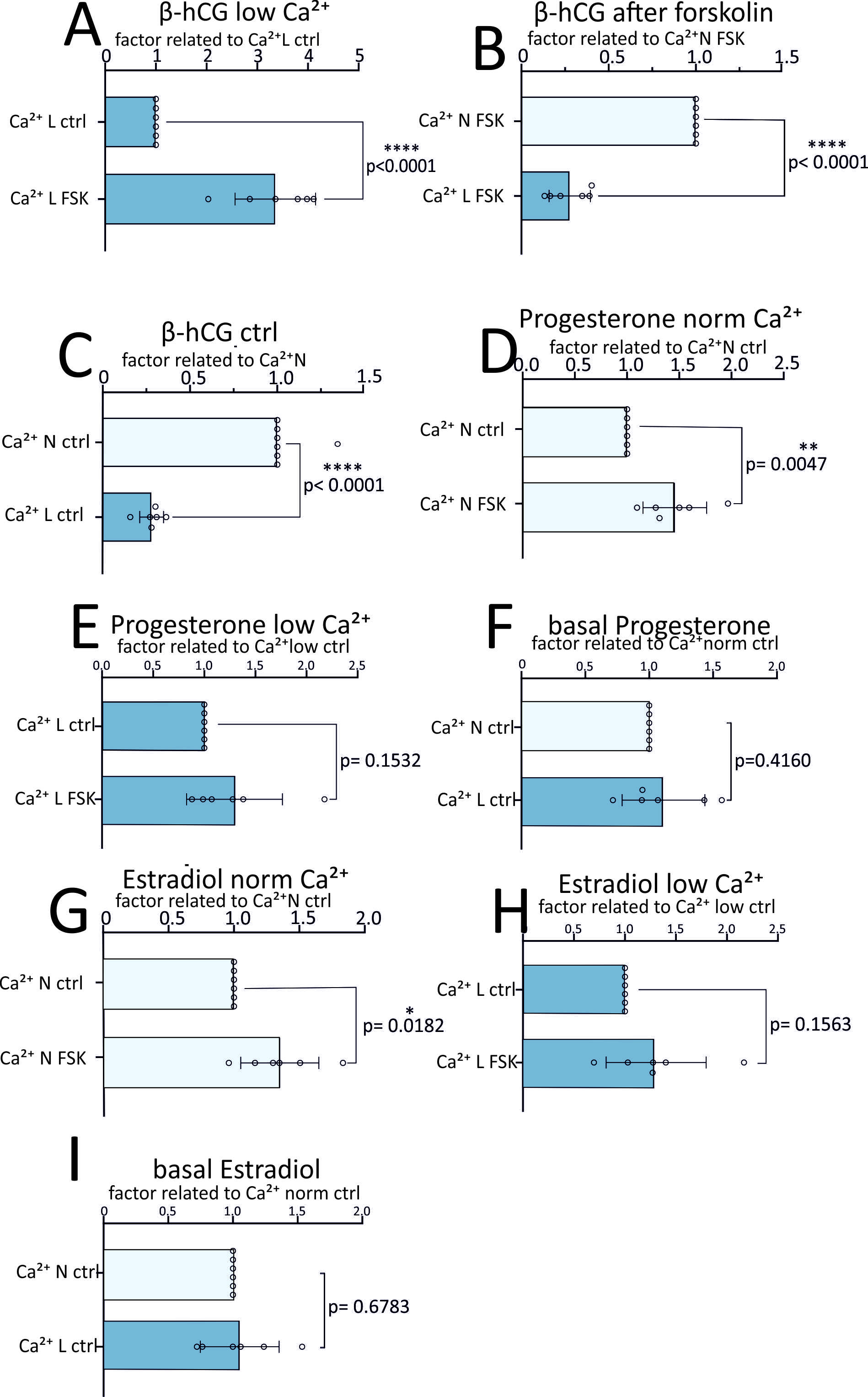

Supplement: Supplementary file 8 — Supplementary Material 8: Supplement Fig. 3: Influence of calcium on steroid hormone secretion of BeWo cells in cell culture supernatant. Cells were stimulated with 30µM FSK. DMSO was used as negative control. The determined hormone concentration was initially related to 1µg protein and afterwards normalized to reference concentration (factor = 1) within the experiment. Ca2+ L (low) = 0.35 mM, Ca2+ N (normal) = 0.94mM (A-C) β-hCG secretion in different treated cells. Analyzed with unpaired t-test, N = 6 (D-F) Progesterone secretion in different treated cells. Analyzed with unpaired t-test, N = 6 (G-I) Estradiol secretion in different treated cells. Analyzed with unpaired t-test, N = 6. Comparison of hormone secretion of BeWo cells treated with low calcium levels and either FSK or DMSO (A, E, H), of BeWo cells treated with FSK and either 0.94mM or 0.35mM calcium (B), of of BeWo cells treated with DMSO and either 0.94mM or 0.35mM calcium (C, F, I) or of BeWo cells treated with 0.94mM calcium and either FSK or DMSO (D, G). [file 12958_2025_1362_MOESM8_ESM.jpg]

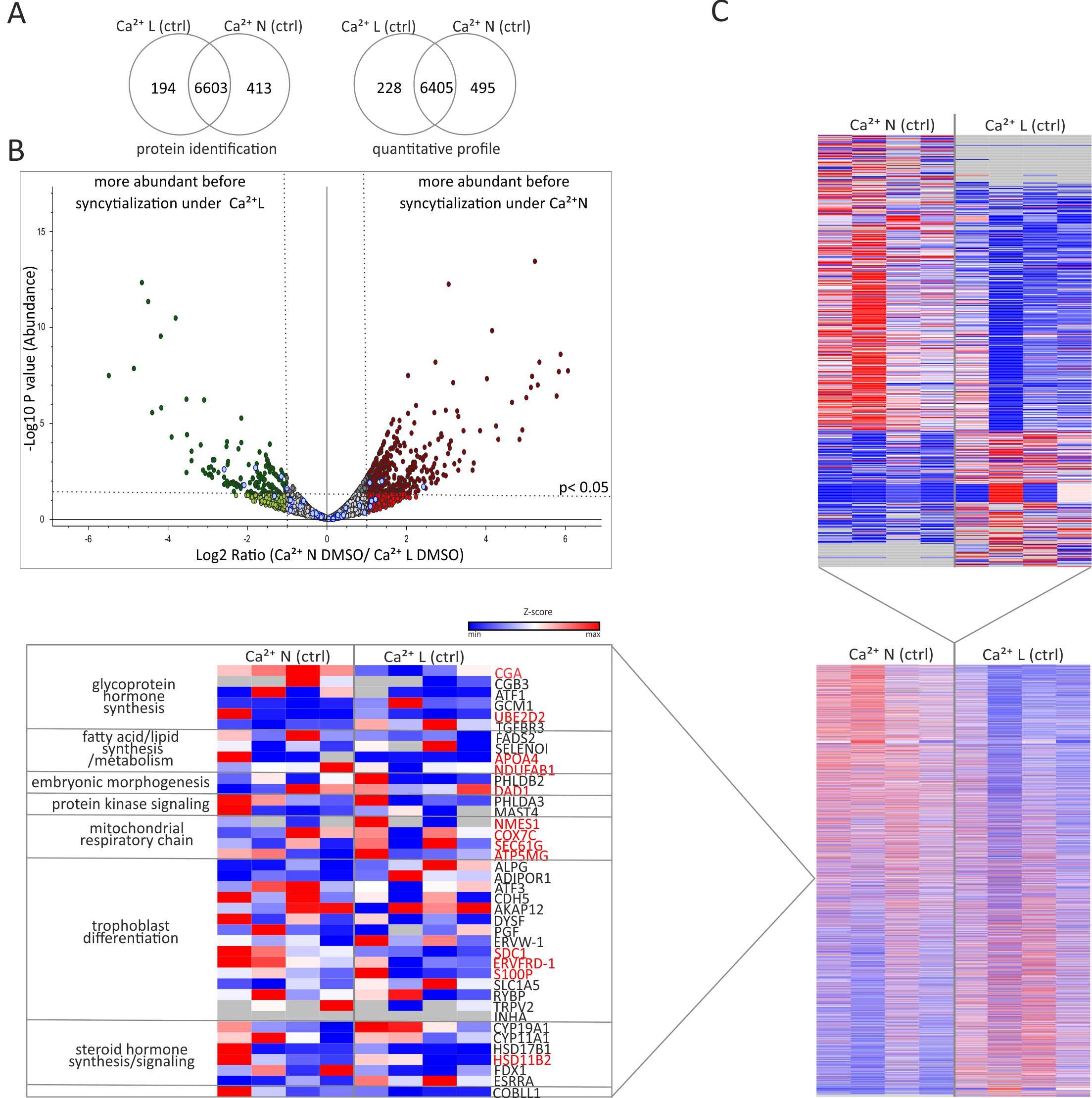

Supplement: Supplementary file 9 — Supplementary Material 9: Supplement Fig. 4: Proteome analysis before syncytialization under normal and under low calcium conditions. (A) Identification and quantification profile of proteome analysis. (B) Volcano plot of quantified proteins in proteome analysis of cytotrophoblast-like cells, N = 4, unpaired t-test. (C) Heatmap of quantified proteins. Lower right side: Heat map of all quantified proteins in proteome analysis of RIPA lysates from unstimulated cytotrophoblast under normal and under low calcium conditions (p < 0.05 and p > 0.05). Upper right side: Heat map of all dysregulated proteins. Lower left side: Heat map of selected proteins due to placental expression and due to classification as part of syncytialization from proteome analysis of stimulated and unstimulated trophoblast-like cells under normal calcium conditions (Fig. 3). [file 12958_2025_1362_MOESM9_ESM.jpg]

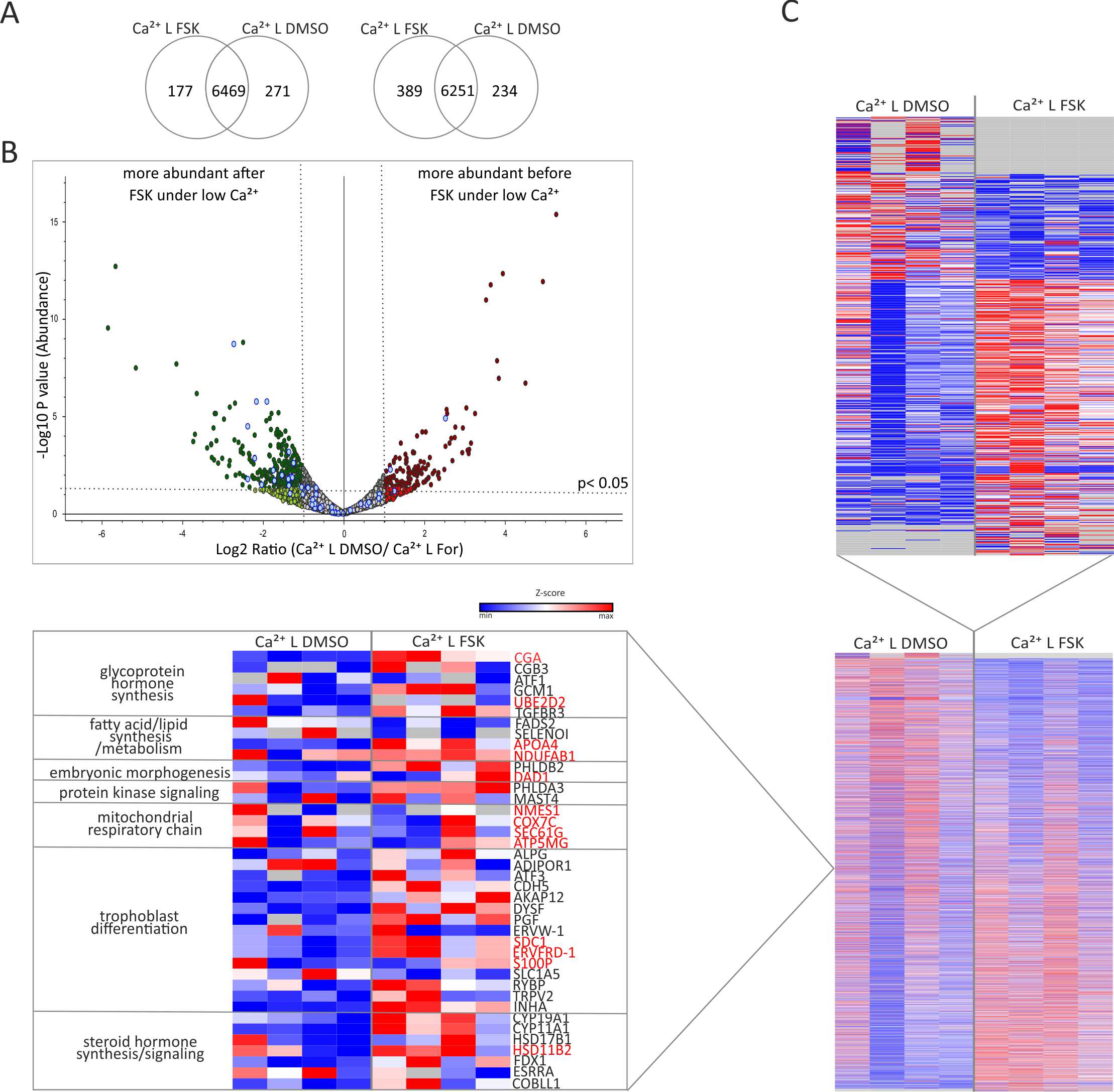

Supplement: Supplementary file 10 — Supplementary Material 10: Supplement Fig. 5: Proteome analysis of undifferentiated and syncytializated trophoblasts under low calcium conditions. (A) Identification and quantification profile of proteome analysis. (B) Volcano plot of quantified proteins in proteome analysis of unstimulated and stimulated trophoblasts, N = 4, unpaired t-test. (C) Heatmap of quantified proteins under low calcium conditions. Lower right side: Heat map of all quantified proteins in proteome analysis (p < 0.05 and p > 0.05). Upper right side: Heat map of all dysregulated proteins. Lower left side: Heat map of selected proteins due to placental expression and due to classification as part of syncytialization from proteome analysis of stimulated and unstimulated trophoblasts under normal calcium conditions (Fig. 3). [file 12958_2025_1362_MOESM10_ESM.jpg]
